# MAP: model-based analysis of proteomic data to detect proteins with significant abundance changes

**DOI:** 10.1038/s41421-019-0107-9

**Published:** 2019-08-13

**Authors:** Mushan Li, Shiqi Tu, Zijia Li, Fengxiang Tan, Jian Liu, Qian Wang, Yuannyu Zhang, Jian Xu, Yijing Zhang, Feng Zhou, Zhen Shao

**Affiliations:** 1 CAS Key Laboratory of Computational Biology, Collaborative Innovation Center for Genetics and Developmental Biology, CAS-MPG Partner Institute for Computational Biology, Shanghai Institute of Nutrition and Health, Shanghai Institutes for Biological Sciences, Chinese Academy of Sciences, Shanghai, 200031 China; 2 University of Chinese Academy of Sciences, Beijing, 100049 China; 3 Department of Immunology and Rheumatology, Gansu Provincial Hospital, Lanzhou, 730000 China; 4 National Laboratory of Plant Molecular Genetics, CAS Center for Excellence in Molecular Plant Sciences, Institute of Plant Physiology and Ecology, Shanghai Institutes for Biological Sciences, Chinese Academy of Sciences, Shanghai, China; 5 Institutes of Biomedical Sciences, Shanghai Medical College of Fudan University, Shanghai, 200032 China; 6 Department of Pediatrics, Children’s Medical Center Research Institute, University of Texas Southwestern Medical Center, Dallas, TX 75390 USA

**Keywords:** Bioinformatics, Proteomic analysis, Proteomics

## Abstract

Isotope-labeling-based mass spectrometry (MS) is widely used in quantitative proteomic studies. With this technique, the relative abundance of thousands of proteins can be efficiently profiled in parallel, greatly facilitating the detection of proteins differentially expressed across samples. However, this task remains computationally challenging. Here we present a new approach, termed Model-based Analysis of Proteomic data (MAP), for this task. Unlike many existing methods, MAP does not require technical replicates to model technical and systematic errors, and instead utilizes a novel step-by-step regression analysis to directly assess the significance of observed protein abundance changes. We applied MAP to compare the proteomic profiles of undifferentiated and differentiated mouse embryonic stem cells (mESCs), and found it has superior performance compared with existing tools in detecting proteins differentially expressed during mESC differentiation. A web-based application of MAP is provided for online data processing at http://bioinfo.sibs.ac.cn/shaolab/MAP.

## Introduction

Over the past two decades, mass spectrometry (MS) has become one of the most powerful tools to quantify protein abundance in biological samples, providing a routine way to analyze protein expression levels and posttranslational modifications^1–4^. Driven by the increasing demand to comprehensively assess protein expression changes across different biological contexts, many MS-based techniques have been developed for relative quantification of protein abundance^1,5–10^. Among them, protein quantification based on stable isotope labeling plays an important role in proteomic studies, largely due to the high accuracy and efficiency^5,8,9^. Most of the isotope labeling-based methods are achieved by introducing different stable isotope labeling into proteins/peptides of different biological samples through metabolic (stable isotope labeling by amino acids in cell culture (SILAC), stable isotope labeling of mammals (SILAM), etc.) or chemical progresses (iTRAQ (isobaric tag for relative and absolute quantitation (iTRAQ)), tandem mass tag (TMT), isotope-coded affinity tag (ICAT), etc.), to create specific mass tags, which can be distinguished by high-resolution MS instruments^1,6–8^.

Using the isotope-labeling technique, the relative expression levels of thousands of proteins across multiple samples can be efficiently quantified in a single MS experiment^7,8,11^. Then, an important task is to accurately identify proteins with significant expression changes between these samples^12–14^. However, the success of this analysis heavily relies on fathoming the technical variability of experiments. Although great progress has been made by isobaric-labeling technique, it still suffers from the “ratio compression” nature^13,15^. In these experiments, the measured ratios of protein/peptide abundance levels across samples are usually underestimated^15,16^. Several approaches have been proposed to solve this problem either by MS/MS/MS (MS3) or gas-phase cleanup strategies^17,18^. However, these strategies cannot be easily implemented in regular instruments and can eventually affect the sensitivity^19,20^. Another possible solution is to build statistical models that can accurately estimate the contribution of technical variations to the ratio observed for each peptide/protein^21^. With these models, even though the measured ratios may be compromised, it is still possible to distinguish true biological differences from technical variations produced by conventional liquid chromatography MS/MS experiments and reliably detect proteins with significant abundance changes^22,23^. Following this direction, a number of statistical models have been developed^10,11,14,24,25^. These models typically require either prior knowledge or separate experiments to assess the noise level of isobaric experiments^10,11,14,24^. For example, Zhang et al.^11^ utilized technical replicates of the same sample, between which no meaningful biological differences should be expected, to model the technical and systematic errors of iTRAQ experiments, and the obtained model was then used as a reference to perform cross-sample comparison. However, instrumental variations may not be negligible among different experiments^26,27^. Thus, a more direct and intuitive approach without borrowing information from additional technical replicates could greatly facilitate researchers to perform differential expression analysis with their own proteomic data.

Here we present a new computational model, termed Model-based Analysis of Proteomic data (MAP), for this purpose. MAP is designed to statistically compare proteomic profiles generated from different biological samples and directly identify proteins with significant abundance changes. It considers all detected proteins as a mixture of differentially and non-differentially expressed ones, and chooses only those with low-intensity changes between two profiles to model the contribution of technical and systematic errors as a function of protein intensity levels. As the key feature of MAP, a novel step-by-step regression analysis is applied to the selected proteins, which first builds local approximations of the error function and then combines them to fit the global error function. Finally, the error function is used as a reference to estimate the significance of the intensity change observed for each protein.

To validate the effectiveness of this approach, we used DEEP-SEQ MS technique^28^ to extensively perform quantitative proteomic profiling of both undifferentiated and differentiated mouse embryonic stem cells (mESCs), and applied MAP to analyze the protein expression changes during mESC differentiation. As a side-by-side comparison, two existing tools including the method presented in Zhang et al.^11^ and MaxQuant^25^, another widely used tool in proteomic studies, were applied to the same dataset. By comparing with a set of published ribosome profiling data of undifferentiated and differentiated mESCs, we provide evidence that our new approach clearly outperformed the existing tools in detecting the differentially expressed proteins (DEPs). In addition, a web-based application of MAP is provided to facilitate its use by the community (http://bioinfo.sibs.ac.cn/shaolab/MAP).

## Results

### Workflow of MAP

**Fig. 1:**
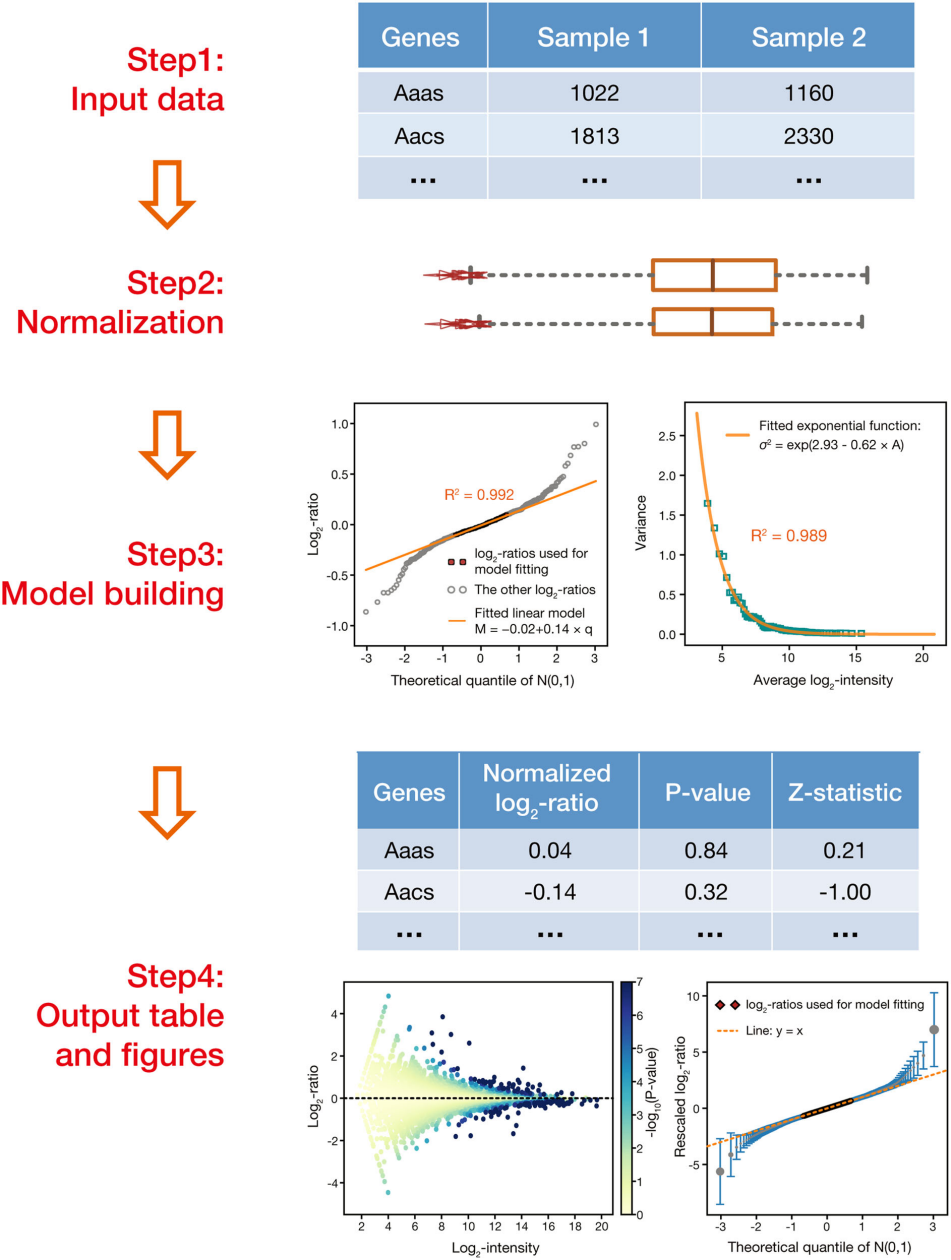
Workflow of MAP. MAP takes two quantitative proteomic profiles generated in the same MS run as input (step 1). After global normalization of MS intensities (step 2), a step-by-step regression analysis is applied to model the contribution of technical and systematic errors to the intensity changes observed (step 3), and the obtained error model is then applied to every detected protein as a reference to infer the significance of its intensity change (step 4)

Figure 1 shows the workflow of MAP, which comprises four main steps. First, it takes two quantitative proteomic profiles generated in the same MS run as input, in which the MS intensities have been assigned to proteins, to represent their relative abundance in each sample. Next, the protein intensities are normalized to make them comparable and the ratio of normalized protein intensities between two profiles is calculated for each protein. Subsequently, in the model building step, a step-by-step regression analysis is applied to the ratios of protein intensities to globally estimate the contribution of technical and systematic errors as a function of protein intensities. Finally, a *P*-value is calculated for each detected protein based on the estimated error function to represent the significance of its abundance change.

To illustrate the performance of MAP, we applied it to compare the proteomic profiles of undifferentiated and differentiated mESCs and detect genes differentially expressed at protein level during mESC differentiation. The proteomics profiling experiments are performed with undifferentiated and differentiated mESCs upon leukemia inhibitory factor (LIF) withdrawal using DEEP-SEQ MS technique^28^. The iTRAQ-based quantitative proteomics experiments were performed three times as biological replicates, which were profiled by three separate MS runs. In general, around 9500 expressed proteins were quantified in each run and most of these identified proteins can be found in multiple runs (Supplementary Fig. S1a and Table S1), indicating a favorable reproducibility between different replicates.

**Fig. 2:**
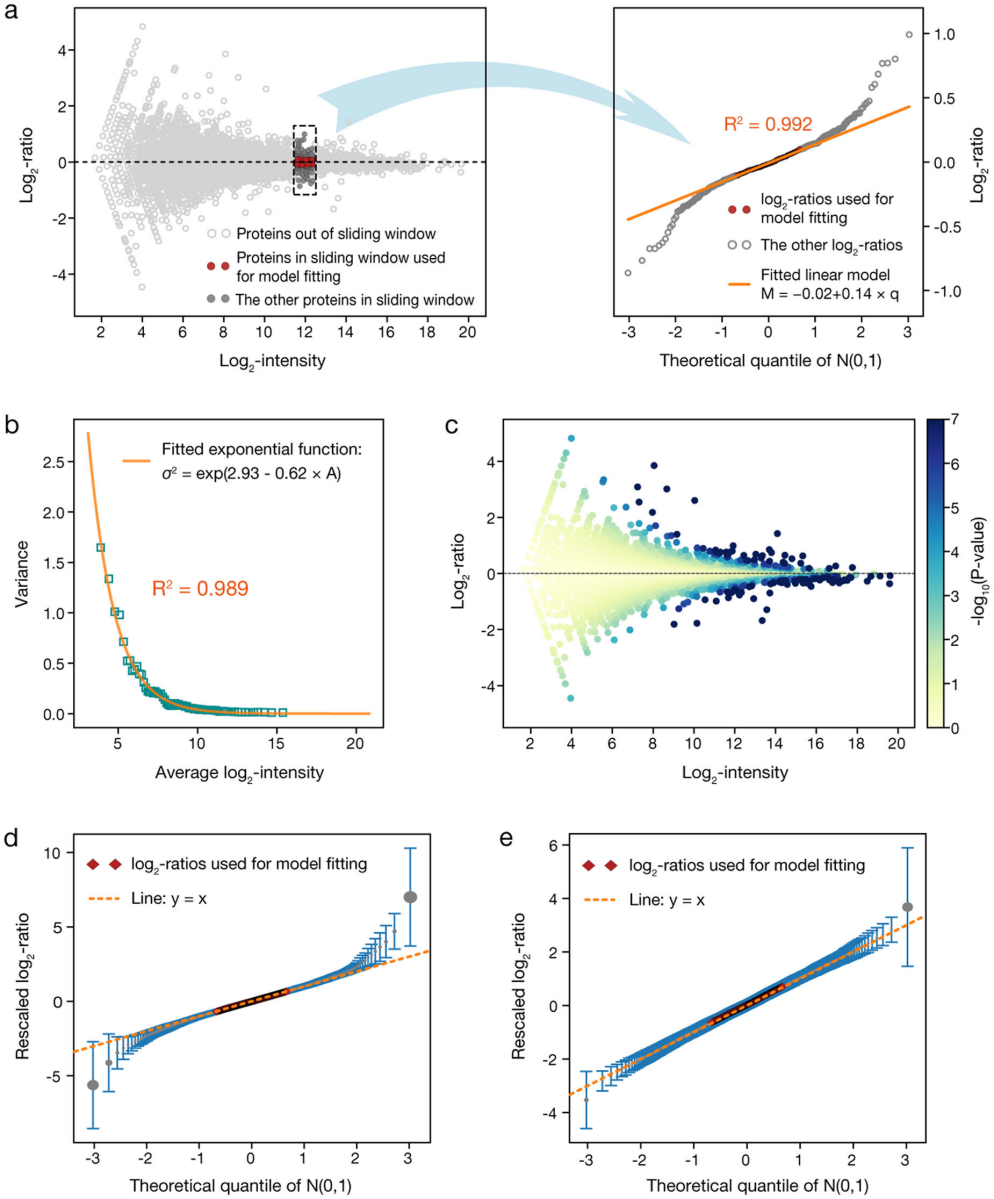
Using MAP to compare the proteomic profiles of undifferentiated and differentiated mESCs. **a** A traditional *MA* plot to show the comparison of the proteomic profiles of undifferentiated and differentiated mESCs generated in the first run (left panel). Here *x* axis is the mean log_2_ intensity of each protein between two profiles (*A*-value) and *y* axis is the log_2_ ratio of protein intensities (*M*-value). A sliding window of size 400 was used to scan the plot with a step size of 100. At each step, all the log_2_ ratios covered by the window were ordered by their values and then plotted against the corresponding theoretical quantiles of standard normal distribution (right panel). The contribution of technical and systematic errors to these log_2_ ratios is assumed to follow approximately the same zero-centered normal distribution *N*(0, *σ*^2^). Next, standard least-square linear regression was applied to the middle 50% of the ordered log_2_ ratios, which were assumed to be predominantly associated with non-differentially expressed proteins, against the corresponding theoretical quantiles to derive a linear model, and the slope of this linear model was taken as an estimation of the parameter σ of the normal distribution for this window (right panel). Here, *R*^2^ is the coefficient of determination of linear regression. **b** The exponential function fitted between the variance *σ*^2^ estimated for each window and the average log_2_ intensities of proteins falling in this window. **c** The *MA* plot shown in **a** with color coding to indicate the *P*-value of each protein’s intensity change. **d** Plot of the ordered log_2_ ratios, which were rescaled by the *σ* estimated for each window and then averaged across all windows, against the corresponding theoretical quantiles of standard normal distribution. Error bars represent the SD from the mean. **e** The same plot as **d**, but here MAP was used to compare the two technical replicates generated in the first run

We started MAP with comparing two proteomic profiles of undifferentiated and differentiated mESCs generated in the first run. First, the protein intensities in two profiles were normalized to make them comparable and then the log_2_ ratio of normalized protein intensities between two profiles was calculated for each protein (Materials and Methods, Supplementary Note 1). To test whether the intensity change observed for each protein is of statistical significance, in MAP we introduced a hypothesis frequently used in differential analysis of proteomic data, in which the contribution of technical and systematic errors to the log_2_ ratio of protein intensities is assumed to follow a zero-centered normal distribution *N*(0, *σ*^2^)^11,21,25^. Here, the variance *σ*^2^ is thought to be a function of the intensity level of each protein^11,25^. In previous studies, this variance function was usually inferred from comparison of technical replicates, which could then be applied to cross-sample comparisons to test whether the observed intensity changes could be explained by technical and systematic errors^11^. To directly build variance function from the two proteomic profiles being compared, in MAP we introduced a novel step-by-step regression analysis. In this analysis, a traditional *MA* plot was first generated^29^, in which the log_2_ ratios of protein intensities of all detected proteins were plotted against their mean log_2_ intensities between two profiles, and then the *MA* plot was scanned with a sliding window from left to right (left panel of Fig. 2a). As the proteins falling in this window have similar intensity levels, the local variance function for these proteins could be approximated by a constant. It is important to note that, when the two profiles being compared are not replicates, these proteins should be considered as a mixture of DEPs and non-DEPs. To infer the local variance function from the non-DEPs in the mixture, the log_2_ ratios of all proteins in this window were ordered by their values and then plotted against the corresponding theoretical quantiles of standard normal distribution *N*(0, 1). In this way, it can be seen that the ordered log_2_ ratios located in the middle, which could be assumed to be mainly associated with non-DEPs, exhibited a strong linear relationship with the corresponding theoretical quantiles (right panel of Fig. 2a). Then, an ordinary least-square linear regression analysis was applied between the middle *W* (the default value for this parameter is *W* = 50%) of the ordered log_2_ ratios and the corresponding theoretical quantiles, and a liner model with coefficient of determination *R*^2^ = 0.992 was derived (right panel of Fig. 2a). This finding indicates that the log_2_ ratios selected for model fitting are predominantly contributed by technical and systematic errors and thus could be thought to be drawn from approximately the same normal distribution, of which the mean and variance could be directly obtained from the parameters of the fitted linear model (see Materials and Methods). The variance of this normal distribution can be taken as a local approximation of the global variance function for proteins covered by this window.

To derive the global variance function, the sliding window was moved in a stepwise manner from left to right. In this way, the whole *MA* plot was covered by a series of windows. At the meantime, the same linear regression analysis was repeatedly applied to each window, to estimate the local variance parameter. Remarkably, the coefficient of determination *R*^2^ was found to be higher than 0.99 for all linear regressions (Supplementary Fig. S2a), strongly supporting the validity of our approach. Next, the variance of the normal distribution estimated by the linear regression applied to each window was plotted against the mean log_2_ protein intensity averaged over proteins falling in this window and an exponential function was fitted between the variance and the mean with coefficient of determination *R*^2^ = 0.989 (Fig. 2b). This exponential function is exactly the global variance function we want to infer for the two profiles under comparison. Finally, a two-tailed *P*-value was calculated for each protein to describe the significance of its intensity change, which was defined as the probability of observing an equal or greater value from normal distribution *N*(0, *σ*^2^) compared with the absolute value of its log_2_ ratio (Fig. 2c). Here, the variance *σ*^2^ was calculated by putting the mean log_2_ intensity of this protein between two profiles into the global variance function (see Materials and Methods).

Furthermore, an additional analysis was carried out in MAP to summarize the local linear regressions. In this analysis, the ordered log_2_ ratios in each window were rescaled by the square root of the variance parameter estimated for this window. Then, the mean and SD of the rescaled log_2_ ratios across all windows were plotted according to their order against the corresponding theoretical quantiles of standard normal distribution (Fig. 2d). This plot in turn could be used to inspect whether the parameter *W* needs to be further adjusted. After rescaling, the middle 50% of the ordered log_2_ ratios were found to match with the line *y* = *x* very well. Meanwhile, the log_2_ ratios close to boundaries obviously deviated from line *y* = *x*, which suggests a considerable fraction of them are associated with DEPs. Thus, they should not be included in inferring the local and global variance function. To validate our hypothesis, we also applied MAP to compare the two technical replicates generated in the first run. In this comparison, the observed protein intensity changes should be contributed by technical and systematic errors. Consistently, we observed that almost all the rescaled log_2_ ratios, even for those not involved in model fitting, could be closely approximated by the line *y* = *x*, except the most extreme ones that might be severely affected by some outlier values (Fig. 2e). Moreover, we repeated this analysis with the proteomic data generated in other runs and got largely similar results (Supplementary Fig. S2b, c). These findings provide a strong support to our hypothesis that the variance function inferred from proteins with low-intensity changes can be extrapolated to all detected proteins as a reference to test whether their intensity changes could be explained by technical and systematic errors.

Inspired by these findings, in MAP we additionally defined a *Z*-statistic for each protein (Fig. 1), which is calculated as the log_2_ ratio of its intensities divided by the square root of the variance *σ*^2^ estimated for it from the global error function. By this means, the contribution of technical errors to each protein’s *Z*-statistic could be assumed to follow the standard normal distribution, independent of its intensity level. Consequently, we found the *Z*-statistics of proteins showed a much higher correlation across different runs than the original log_2_ ratios of protein intensities (Supplementary Fig. S2d, e), suggesting they are more comparable with each other and can provide a better basis for integrating the protein expression changes observed in different runs (Supplementary Note 2).

### Comparison of MAP with existing methods

**Fig. 3:**
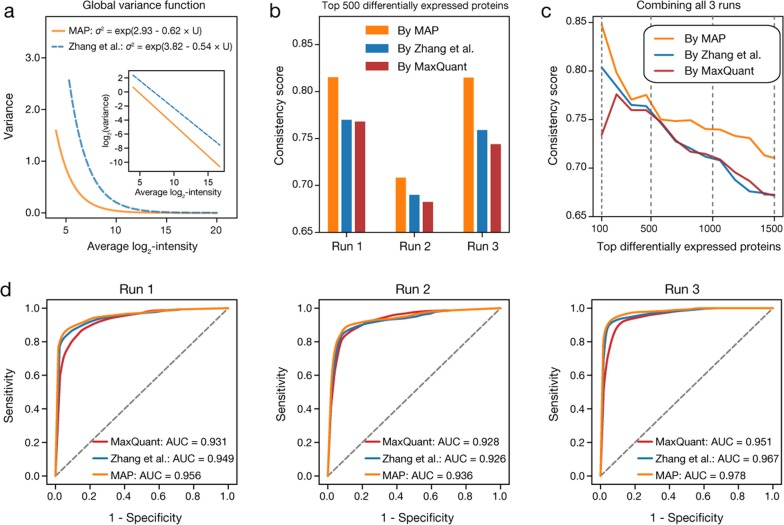
Performance comparison of MAP and two existing methods in detecting proteins differentially expressed during mESC differentiation. **a** The global variance function obtained by using MAP to compare the proteomic profiles of undifferentiated and differentiated mESCs generated in the first run (solid line), as well as that got by applying the method proposed in Zhang et al.^11^ on the two technical replicates generated in the same run (dashed line). **b** The consistency score of the top 500 differentially expressed proteins between undifferentiated and differentiated mESCs detected by MAP, the method proposed in Zhang et al.^11^ as well as MaxQuant from each of the three MS runs. Here the consistency score was defined as the fraction of proteins whose abundance changes are consistent with the translation changes of the corresponding mRNA transcripts detected from ribosome profiling data in terms of direction. **c** The consistency score of the top 500, 1000, and 1500 differentially expressed proteins between undifferentiated and differentiated mESCs detected by MAP, the method presented in Zhang et al.^11^ as well as MaxQuant from all three runs. Here, the top differentially expressed proteins were selected based on the most significant *P*-value of each protein among all three runs. **d** The receiver operating characteristic (ROC) curves of using the *P*-values derived by different methods in each single run to distinguish the benchmark differentially expressed proteins (DEPs) from other proteins and the area under each ROC curve (AUC) was also calculated to indicate the performance of each method

MAP directly uses the two proteomic profiles under comparison to model the impact of technical and systematic errors, as represented by the global variance function, and identifies proteins with significant abundance changes based on it. In contrast, many existing methods require additional technical replicates to build reference error model^10,11,14,30^. We took the global variance functions obtained by applying MAP to directly compare the proteomic profiles of differentiated and undifferentiated mESCs, and compared them with the variance functions derived from parallel technical replicates generated in the same MS run by using the method described in Zhang et al.^11^. Interestingly, the variance functions obtained by these two methods showed considerable difference (Fig. 3a and Supplementary Fig. S3a-b). Therefore, it is necessary to assess which method could provide a more reliable estimation of the statistical significance of observed protein abundance changes. Moreover, we included another commonly used tool, MaxQuant^25^, which also can directly perform statistical comparison between different proteomic profiles. MaxQuant does not model the impact of technical errors as a function of protein intensities, but simply uses the 15.87, 50, and 84.13th percentiles of the global and local distribution of protein ratios to detect proteins with significant outlier ratios^25^.

We applied all three methods to the proteomic data of differentiated and undifferentiated mESCs generated in each run, and selected the same number of top DEPs for each method based on the *P*-values calculated by it. Then, we used the published ribosome profiling data of differentiated and undifferentiated mESCs as a reference^31^ to evaluate the reliability of the DEPs detected by each methods. For each set of DEPs, we compared their abundance changes with the mRNA translation changes of the corresponding genes obtained from ribosome profiling data and defined the consistency score of this set of proteins as the fraction of them exhibiting consistent changes in terms of direction (see Materials and Methods). In this way, the top 500 DEPs detected by MAP were found to have an obviously higher consistency score than those identified by the other two methods for all three runs (Fig. 3b). Moreover, we assigned each protein with the most significant *P*-value of all three runs and then selected the top 500, 1000, and 1500 DEPs for each method. Again, the DEPs detected by MAP achieved the highest consistency score (Fig. 3c).

In addition, we conducted an analysis using benchmark DEPs between undifferentiated and differentiated mESCs defined from the proteomic data under comparison to further assess the performance of three different methods, and found MAP showed a better sensitivity and specificity in recovering the benchmark DEPs from the comparison of proteomic profiles generated in each single run than the other two methods (Fig. 3d and Supplementary Fig. S4a–c, and Note 4). Taken together, these results suggest MAP not only can increase the efficiency of quantitative proteomic study but also has improved performance in detecting proteins with significant abundance changes.

### Web-based application of MAP

**Fig. 4:**
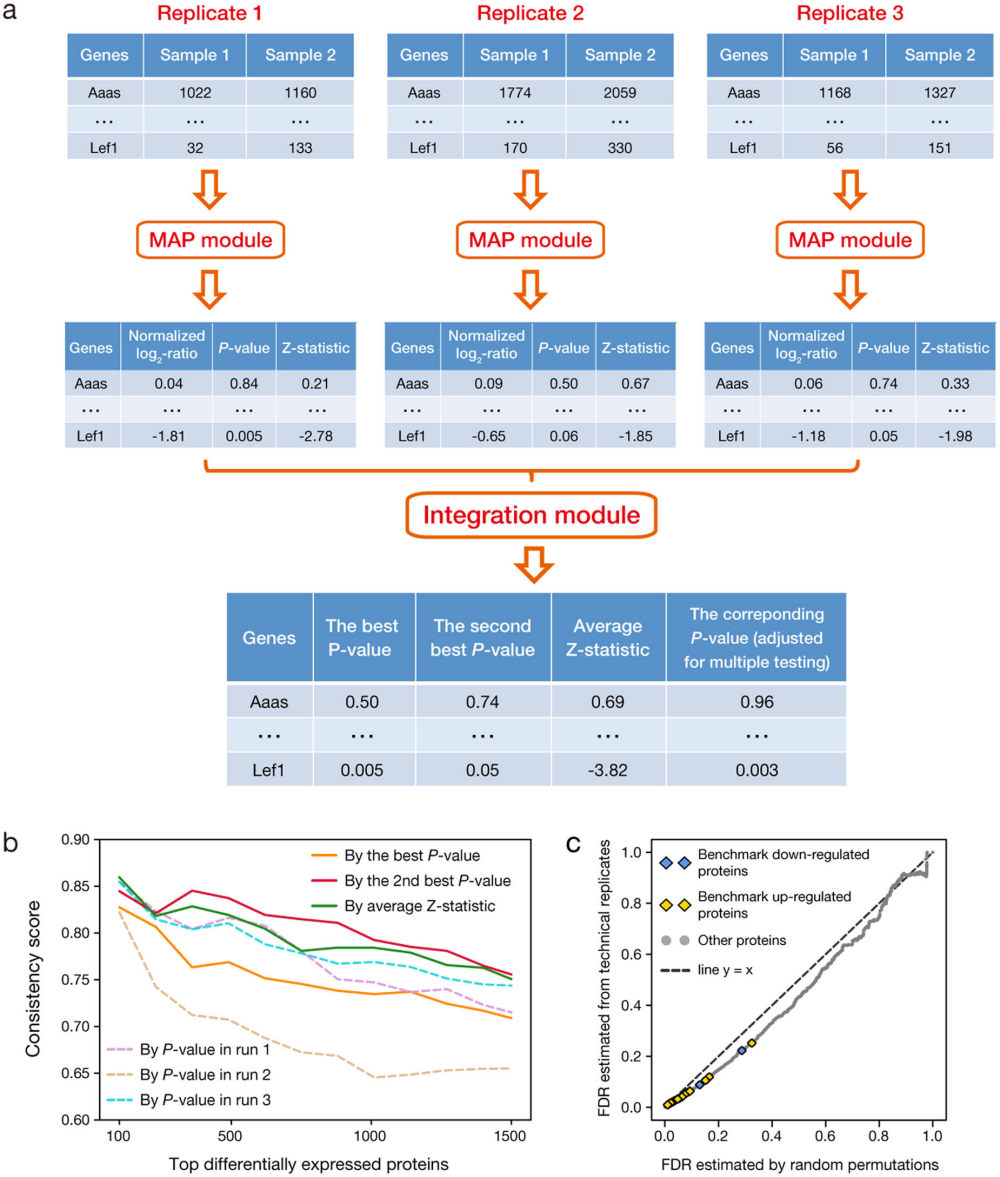
Using MAP to handle proteomic data with biological replicates. **a** Workflow of using the web application of MAP to compare proteomic data with multiple biological replicates generated in different MS runs. **b** The consistency score of the top differentially expressed proteins between undifferentiated and differentiated mESCs identified by MAP. Here, proteins were ranked by the best *P*-value (blue solid line), the second best *P*-value (red solid line), and the average *Z*-statistic (green solid line) among all three runs, as well as by the *P*-value of each run, respectively (dashed lines). **c** False discovery rate (FDR) estimated for the second best *P*-value of each protein using the permutation-based approach were plotted against that estimated from the comparisons of technical replicates for this second best *P*-value. Here, FDRs associated with the benchmark DEPs defined previously were explicitly indicated

We provide a web-based application of MAP (http://bioinfo.sibs.ac.cn/shaolab/MAP) to facilitate its use by researchers with no programming experience. Moreover, in many studies the proteomic profiling experiments were performed for multiple times as biological replicates, which can increase both the coverage of proteomic data and also the reproducibility of the results^28,32^. Our web-based application of MAP allows users to perform a two-step differential analysis on their own proteomic data with multiple replicates, which may be generated in different MS runs (Fig. 4a). At the first step, they can use the MAP module to compare the proteomic profiles generated in each run separately. Then, the output statistics of all replicates can be combined at the integration module to finally determine the DEPs. Here, two alternative approaches are provided for data integration. We first borrowed the concept of the second best rule frequently used for high-throughput short hairpin RNA (shRNA) screening^33–36^. In those studies, typically, multiple shRNAs were designed for each single gene and the candidate genes were ranked by the second best score of the shRNAs against each gene to increase the confidence of ranking. Besides, some users may also be interested in the overall expression changes across replicates, e.g., when the proteomic profiling experiments are performed with patient samples or the number of replicates is large^37,38^. Inspired by the Stouffer’s *Z*-test^39^, the integration module additionally calculates the average *Z*-statistic of each protein over all replicates as well as the corresponding *P*-value based on the standard normal distribution (see Materials and Methods), providing another way to summarize the protein expression changes detected (Fig. 4a).

To test the performance of these methods on proteomic data integration, we repeatedly used MAP to compare the proteomic profiles of undifferentiated and differentiated mESCs generated in each run, and then ranked all detected proteins by the best and the second best *P*-value, as well as the average *Z*-statistic of each protein over all three runs. By this analysis, we observed that the top DEPs selected based on the second best *P*-value and average *Z*-statistic have clearly better consistency scores than those selected by the best *P*-value of three runs, and are also better than those identified in any single run (Fig. 4b). This analysis again highlights the importance of having biological replicates for quantitative proteomic studies. Moreover, inspired by the analysis presented in two previous studies^40,41^, we developed a permutation-based approach to estimate the false discovery rate (FDR) for the second best *P*-value and the average *Z*-statistic of each protein (Supplementary Note 5 and Fig. S5a–c), which represents the estimated type-I error rate for the DEPs defined by using each of them as cutoff, and provided in the integration module. As the result, we found FDRs estimated by the permutation-based method agreed well with those estimated from technical replicates (Fig. 4c and Supplementary Fig. S5d), indicating the validity of this approach.

## Conclusion and discussion

In this work, we present a new computational tool, MAP, to statistically compare proteomic data generated using isotope-labeling-based MS technique and evaluate the significance of the abundance change detected for each protein. Unlike many existing methods for this purpose, MAP does not rely on technical replicates to model technical and systematic errors and, instead, directly builds error model from the proteomic profiles under comparison using a step-by-step regression analysis. This improvement can increase the efficiency of MS experiments. Moreover, considerable differences between the error models built by two different classes of approaches are still noted, even though technical replicates generated in the same MS run were used for model fitting. Therefore, we applied MAP and two existing tools to compare the proteomic profiles of undifferentiated and differentiated mESCs, and found that the protein expression changes detected by MAP exhibited a clearly better consistency with the changes of mRNA translation detected from corresponding ribosome profiling data, indicating a favorable performance of our new approach. As a natural extension of MAP model, we found that, by using the sample variance of each protein’s log_2_ intensities and the *χ*^2^-distribution to replace the log_2_ ratio of protein intensities and the standard normal distribution used in the step-by-step regression procedure of MAP, respectively, a new statistical framework can be derived for simultaneously comparing multiple proteomic profiles generated in the same MS run and identifying proteins differentially expressed across them (Supplementary Fig. S6a–f, Table S2, and Note 6).

Furthermore, it will be important to note the high consistency observed between the protein expression changes and the mRNA translation changes during mESC differentiation. This finding provides a new insight into posttranscriptional regulation of gene expressions. For example, close to 85% of the top 500 DEPs detected by MAP showed parallel changes of mRNA translation, and this fraction for the top 1500 DEPs is still higher than 75% (Fig. 4b). Given the fact that these proteins typically are highly abundant in the samples being compared (Fig. 2c), it is reasonable to speculate that a large fraction of the posttranscriptional regulation of gene expressions, especially for those highly expressed genes, may be mediated by RNA sequence signatures, which can also be supported by the findings of many recent studies^42–44^. Thus, a systematic and integrative analysis of proteomic, transcriptomic, and ribosome profiling data can provide critical information about the mechanism of posttranscriptional regulation of gene expression.

## Materials and methods

### Cell culture and proteomic sample preparation

The J1 mESCs were lysed in the SDS solution (50 mM Tris-HCl, pH 7.5, 5% SDS, 100 mM DTT, 5 mM EDTA). After boiling in water for 20 min, the extracted proteins were precipitated by adding six volumes of cold (−20 °C) acetone and resolubilized in a digestion buffer containing 8 M urea and 0.1 M NH_4_HCO_3_. Dithiothreitol (DTT) was added to a final concentration of 100 mM and incubated for 30 min at 37 °C, and then 20 mM iodoacetamide was added and incubated in the dark at room temperature for 30 min; excess iodoacetamide was quenched by addition of DTT to a final concentration of 20 mM. The proteins were then diluted to a final urea concentration of 1 M urea with 0.1 M ammonium bicarbonate. Trypsin digestion was performed at 37 °C overnight with end-over-end rotation. The digested peptide solution was acidified with 1% trifluoroacetic acid (TFA) and desalted on a C18 solid-phase extraction cartridge 96-well plates. Eluted peptides were lyophilized by vacuum centrifugation andstored at −80 °C in 400 µg aliquots.

To compare the proteome of undifferentiated and differentiated mESCs, peptides from the mESCs incubated without LIF for 48 h were labeled as two technical replicates with iTRAQ 116 and 117. The peptides from the undifferentiated mESCs incubated with LIF were labeled as technical replicates with iTRAQ 114 and 115. For each reaction, peptides were solubilized in 500 mM triethylammonium bicarbonate and mixed with the iTRAQ reagent in ethanol. Labeling was performed at room temperature for 1 h and samples were then combined. Fresh aliquots of mESC were processed as described above, to provide biological triplicates.

The protein quantification was performed with a DEEP-SEQ platform described previously^28^. Briefly, the iTRAQ-labeled peptides was solubilized in the running buffer (10 mM ammonium formate at pH 10) and loaded onto the first dimension column packed with C18 (high pH RP) resin. The peptides were fractionated and then loaded onto the second dimension column packed with strong anion exchange (SAX) resin. Afterward, the fractionated peptides were further separated in SAX column and finally processed in a 25 μm I.D. column, and introduced into a Triple-TOF 5600 mass spectrometer (Sciex Framingham, MA). The 5600 mass spectrometer was operated in data-dependent mode, with the top 50 precursors (charge state +2 to +5, >100 counts) in each MS scan (800 ms, scan range 350–1400 m/z) subjected to MS/MS (minimum time 140 ms, scan range 100–1400 m/z). A dynamic exclusion window of 40 s was used with unit resolution for precursor isolation. Electrospray voltage was set to 2.4 kV. The result wiff files were processed with proteinpilot v4.5 (sciex) to search against human database downloaded from uniprot. All peptide spectral matches (PSMs) from three biological replicates were combined for the FDR assessment. Only those peptides with scores at or above a PSM FDR threshold of 1% were further considered.

### Normalization of protein intensities

In this study, the iTRAQ intensities of peptides representing their relative abundance have already been summarized to protein level before sending to MAP for comparison. Before carrying out differential protein expression analysis between undifferentiated and differentiated mESCs, protein intensities from channel 114 and 115 in each run were added together to generate the proteomic profile of undifferentiated mESCs, and those from channel 116 and 117 were combined as the proteomic profile of differentiated mESCs (Supplementary Note 3 and Fig. S2f). For each profile, the outlier proteins are identified as those with intensities higher than *Q*_3_ + *L* * (*Q*_3_ − *Q*_1_), where *Q*_1_ and *Q*_3_ are the 25th and 75th percentiles (i.e., the lower and upper quartiles) of the protein intensities in this profile, respectively, and *L* = 1.5 is used in this study. Then, the trimmed total intensity of each profile is calculated as the sum of protein intensities over proteins that are not identified as outliers in the profiles under comparison. Finally, protein intensities in each profile are divided by the corresponding normalization factor, which is calculated as the trimmed total intensity of this profile divided by the average trimmed total intensity of the profiles being compared. An evaluation of the normalization of protein intensities based on trimmed total intensities can be found in Supplementary Note 1, Fig. S1b, c, and Fig. S3c, d.

### Calculation of *P*-value to characterize the significance of each protein’s intensity change

For protein *i*, its average intensity between the two profiles being compared is calculated as

$$A_i = \frac{1}{2}\left( {\text{log}_2S_{i1}} + {\text{log}_2S_{i2}} \right),$$

where *S*_*i*1_ and *S*_*i*2_ are the intensity of this protein in the first and second profile, respectively, and the log_2_ ratio of its protein intensities is calculated as

$$M_i = {\text{log}}_2\left( {\frac{{S_{i1}}}{{S_{i2}}}} \right)$$

In MAP, the contribution of technical and systematic errors to *M*_*i*_ is assumed to follow a zero-centered normal distribution *N*(0, *σ*_*i*_^2^) and *σ*_*i*_^2^ is modeled as a function of *A*_i_. To directly infer this variance function from the profiles being compared, the log_2_ ratios of all detected proteins are plotted against their average intensities to generate a traditional *M*–*A* plot and the *M*–*A* plot is then scanned by a sliding window of size *N* proteins moving from left to right (*N* = 400 and step size = 100 here). In this way, the whole plot is covered by a series of windows, which are used to first build local estimations of the global variance function. Here, MAP introduces an assumption that proteins falling in each window have similar intensity levels and thus the contribution of technical and systematic errors in the log_2_ ratios falling in each window can be assumed to follow approximately the same normal distribution *N*(0, *σ*^2^). To estimate the variance parameter *σ*^2^ for each window, all the log_2_ ratios covered by this window are ordered by their values and then plotted against the corresponding theoretical quantiles of standard normal distribution. Here, the plotting position $\hat p_i$ (i.e., the choice of theoretical quantile) associated with the *i*-th log_2_ ratio is calculated based on the formula proposed by Michael and Schucany in 1986^45^ for censored data

$$\hat p_i = \frac{{N - a + 1}}{{N - 2a + 1}}\mathop {\prod}\limits_{j \subset \Omega {\mathrm{,}}j \ge i} {\frac{{j - a}}{{j - a + 1}}}$$

(here Ω is the set of data points involved in the corresponding analysis, which, e.g., is defined as the log_2_ ratios in the middle selected for linear regression), and then the corresponding theoretical quantile of standard normal distribution $\hat q_i = \Phi ^{ - 1}\left( {\hat p_i} \right)$ can be derived from the reverse function of

$$\hat p_i = \Phi \left( {\hat q_i} \right) = {\int_{-\infty }^{\hat q_i}} {\frac{1}{{\sqrt {2\pi } }}} \ast e^{ - x^2/2}dx$$

Next, an ordinary least-square linear regression is applied to the middle *W* (*W* = 50% by default) of the ordered log_2_ ratios in each window against the corresponding theoretical quantiles to derive a linear model

$$M = {\mathrm{\mu }} + {\mathrm{\sigma }} \ast \hat q,$$

and the square of the slope *σ* is used to as an estimation of the variance *σ*^2^ for this window. When the sliding window finishes scanning the *MA* plot, nonlinear least-square regression is applied to fit a two-parameter exponential function between the variance *σ*^2^ estimated for each window and the average protein intensity *A* over the proteins selected for linear regression in this window as

$${\mathrm{\sigma }}^2 = \Psi \left( {\theta ,A} \right) = \exp \left( {\theta _1 + \theta _2 \cdot {\mathrm{A}}} \right)$$

This exponential function is the global variance function for the two profiles being compared. Finally, a two-tailed *P*-value is calculated for each protein to represent the significance of its intensity change, as the probability of observing an equal or greater value than the absolute value of its log_2_ ratio *A*_*i*_ from normal distribution ${\mathrm{N}}\left( {0,{\mathrm{\sigma }}_i^2 = \Psi \left( {\theta ,A_i} \right)} \right)$ using formula

$$P_i = 2 \ast {\int_{|{\mathrm{M}}_i|}^{ + \infty }} {\frac{1}{{\sqrt {2\sigma _i\pi } }}} \ast e^{ - x^2/(2\sigma _i^2)}dx,$$

which will be further adjusted for multiple testing using the Benjamini–Hochberg approach.

In addition, the mean value of the log_2_ ratios falling in each window, as estimated by *μ* of the linear model derived from regression, often fluctuates slightly around zero. This observation could be explained by the local bias of global protein intensity normalization and it is extremely hard to be completely removed. MAP provides user with an option to calculate the *P*-values in a more stringent way. In this option, MAP considers the contribution of local bias of normalization to the log_2_ ratio observed for each protein by adding a constant term ${\mathrm{\sigma }}_\mu ^2$ to the variance of the reference normal distribution ${\mathrm{N}}(0,{\mathrm{\sigma }}_i^2)$ used for *P*-value calculation as ${\mathrm{\sigma }}_i^2 = \Psi \left( {\theta ,A_i} \right) + {\mathrm{\sigma }}_\mu ^2$, in which ${\mathrm{\sigma }}_\mu ^2$ is the variance of parameter *μ* across all windows.

### Definition of the *Z*-statistic of each protein’s intensity change

In the comparison of two proteomic profiles using MAP, the *Z*-statistic of protein *i* is defined as

$${Z}_i = \frac{{{M}_i}}{{{\mathrm{\sigma }}_i}}.$$

Furthermore, the average Z-statistic of protein *i* across multiple comparisons is defined as

$${\hat{Z}}_i = \frac{{\mathop {\sum }\nolimits_{t = 1}^k Z_i^t}}{{\sqrt k }},$$

and here k is the number of comparisons in which protein *i* is detected in the proteomic profiles being compared. Next, a two-tailed *P*-value is calculated for the average Z-statistic of this protein based on standard normal distribution

$$P = 2 \ast {\int_{|{\hat{\mathrm Z}}_i|}^{ + \infty}} {\frac{1}{{\sqrt {2\pi } }}} \ast e^{ - x^2/2}dx,$$

which will also be adjusted using the Benjamini–Hochberg approach for multiple testing.

### Integration with ribosome profiling data

The translation changes of mouse genes during mESC differentiation after LIF withdrawal based on ribosome profiling experiments were directly downloaded from Ingolia et al.^31^. Only genes with log_2_ ratios of translation lower than −0.2 or higher than 0.2 were retained. In this way, 2709/3192 genes with increased/decreased mRNA translation during mESC differentiation were used for the following analysis, respectively. Next, the consistency score of each set of DEPs is defined as

$${\mathrm{Consistency}}\;{\mathrm{score}} = \frac{{\# {\mathrm{Consistent}}\;{\mathrm{DEPs}}}}{{\# {\mathrm{Consistent}}\;{\mathrm{DEPs}} + \# {\mathrm{Inconsistent}}\;{\mathrm{DEPs}}}}$$

Here, the consistent DEPs are defined as DEPs whose direction of abundance changes is consistent with the direction of translation changes of corresponding genes, and inconsistent DEPs are defined as those showing opposite changes in terms of direction.

## Supplementary information

Supplementary information, notes and Figures.

Supplementary information, Table S1

Supplementary information, Table S2

## Supplementary information

**Supplementary Information** accompanies the paper at (10.1038/s41421-019-0107-9).

## References

[1] Gygi SP (1999). Quantitative analysis of complex protein mixtures using isotope-coded affinity tags. Nat. Biotechnol..

[2] Oda Y, Huang K, Cross FR, Cowburn D, Chait BT (1999). Accurate quantitation of protein expression and site-specific phosphorylation. Proc. Natl Acad. Sci. USA.

[3] Paša-Tolić L (1999). High throughput proteome-wide precision measurements of protein expression using mass spectrometry. J. Am. Chem. Soc..

[4] Aebersold R, Mann M (2003). Mass spectrometry-based proteomics. Nature.

[5] Yao X, Freas A, Ramirez J, Demirev PA, Fenselau C (2001). Proteolytic 18O labeling for comparative proteomics: model studies with two serotypes of adenovirus. Anal. Chem..

[6] Ong SE (2002). Stable isotope labeling by amino acids in cell culture, SILAC, as a simple and accurate approach to expression proteomics. Mol. Cell. Proteomics.

[7] Thompson A (2003). Tandem mass tags: a novel quantification strategy for comparative analysis of complex protein mixtures by MS/MS. Anal. Chem..

[8] Ross PL (2004). Multiplexed protein quantitation in Saccharomyces cerevisiae using amine-reactive isobaric tagging reagents. Mol. Cell. Proteomics.

[9] Schmidt A, Kellermann J, Lottspeich F (2005). A novel strategy for quantitative proteomics using isotope-coded protein labels. Proteomics.

[10] Zhou C (2014). A hierarchical statistical modeling approach to analyze proteomic isobaric tag for relative and absolute quantitation data. Bioinformatics.

[11] Zhang Y (2010). A robust error model for iTRAQ quantification reveals divergent signaling between oncogenic FLT3 mutants in acute myeloid leukemia. Mol. Cell. Proteomics.

[12] Karp NA (2010). Addressing accuracy and precision issues in iTRAQ quantitation. Mol. Cell Proteomics.

[13] Mertins P (2012). iTRAQ labeling is superior to mTRAQ for quantitative global proteomics and phosphoproteomics. Mol. Cell. Proteomics.

[14] Breitwieser FP (2011). General statistical modeling of data from protein relative expression isobaric tags. J. Proteome Res..

[15] Ow SY (2009). iTRAQ underestimation in simple and complex mixtures: “the good, the bad and the ugly”. J. Proteome Res..

[16] Ting L, Rad R, Gygi SP, Haas W (2011). MS3 eliminates ratio distortion in isobaric multiplexed quantitative proteomics. Nat. Methods.

[17] Konishi Y (2007). Molecular formula analysis by an MS/MS/MS technique to expedite dereplication of natural products. Anal. Chem..

[18] Geromanos SJ (2009). The detection, correlation, and comparison of peptide precursor and product ions from data independent LC-MS with data dependant LC-MS/MS. Proteomics.

[19] Kind T, Fiehn O (2010). Advances in structure elucidation of small molecules using mass spectrometry. Bioanalytical Rev..

[20] Kingston DG (2011). Modern natural products drug discovery and its relevance to biodiversity conservation. J. Nat. Prod..

[21] Jorge I (2009). Statistical model to analyze quantitative proteomics data obtained by 18O/16O labeling and linear ion trap mass spectrometry: application to the study of vascular endothelial growth factor-induced angiogenesis in endothelial cells. Mol. Cell. Proteomics.

[22] Zenón F (2016). 18O proteomics reveal increased human apolipoprotein CIII in Hispanic HIV‐1+ women with HAART that use cocaine. Proteomics Clin. Appl..

[23] Husain A (2016). Chromatin remodeller SMARCA4 recruits topoisomerase 1 and suppresses transcription-associated genomic instability. Nat. Commun..

[24] Mandel M, Askenazi M, Zhang Y, Marto JA (2013). Variance function estimation in quantitative mass spectrometry with application to iTRAQ labeling. Ann. Appl. Stat..

[25] Cox J, Mann M (2008). MaxQuant enables high peptide identification rates, individualized p.p.b.-range mass accuracies and proteome-wide protein quantification. Nat. Biotechnol..

[26] Wu L (2005). Quantitative analysis of the microbial metabolome by isotope dilution mass spectrometry using uniformly 13 C-labeled cell extracts as internal standards. Anal. Biochem..

[27] Kume H (2014). Discovery of colorectal cancer biomarker candidates by membrane proteomic analysis and subsequent verification using selected reaction monitoring (SRM) and tissue microarray (TMA) analysis. Mol. Cell. Proteomics.

[28] Zhou F (2013). Genome-scale proteome quantification by DEEP SEQ mass spectrometry. Nat. Commun..

[29] Yang Y. H. (2002). Normalization for cDNA microarray data: a robust composite method addressing single and multiple slide systematic variation. Nucleic Acids Research.

[30] Zhou C (2012). Statistical considerations of optimal study design for human plasma proteomics and biomarker discovery. J. Proteome Res..

[31] Ingolia NT, Lareau LF, Weissman JS (2011). Ribosome profiling of mouse embryonic stem cells reveals the complexity and dynamics of mammalian proteomes. Cell.

[32] Evans C (2012). An insight into iTRAQ: where do we stand now?. Anal. Bioanal. Chem..

[33] Chudnovsky Y (2014). ZFHX4 interacts with the NuRD core member CHD4 and regulates the glioblastoma tumor-initiating cell state. Cell Rep..

[34] Whittaker SR (2013). A genome-scale RNA interference screen implicates NF1 loss in resistance to RAF inhibition. Cancer Discov..

[35] Keenan MM (2015). ACLY and ACC1 regulate hypoxia-induced apoptosis by modulating ETV4 via alpha-ketoglutarate. PLoS Genet..

[36] Luo B (2008). Highly parallel identification of essential genes in cancer cells. Proc. Natl Acad. Sci. USA.

[37] Zhang H (2016). Integrated proteogenomic characterization of human high-grade serous ovarian. Cancer. Cell.

[38] Mertins P (2016). Proteogenomics connects somatic mutations to signalling in breast cancer. Nature.

[39] Whitlock MC (2005). Combining probability from independent tests: the weighted Z-method is superior to Fisher's approach. J. Evol. Biol..

[40] Xie Y, Pan W, Khodursky AB (2005). A note on using permutation-based false discovery rate estimates to compare different analysis methods for microarray data. Bioinformatics.

[41] Jiao S, Zhang S (2008). On correcting the overestimation of the permutation-based false discovery rate estimator. Bioinformatics.

[42] Izquierdo JM, Cuezva JM (2000). Internal-ribosome-entry-site functional activity of the 3'-untranslated region of the mRNA for the beta subunit of mitochondrial H+-ATP synthase. Biochem. J..

[43] Thoreen CC (2012). A unifying model for mTORC1-mediated regulation of mRNA translation. Nature.

[44] Liu X (2017). Regulation of mitochondrial biogenesis in erythropoiesis by mTORC1-mediated protein translation. Nat. Cell Biol..

[45] Michael, J. R. & Schucany, W. R. Analysis of data from censored samples *Goodness-of-Fit Techniques* 461–496 (Marcel Dekker: New York, 1986).

