# Supplementary material for: MAP: model-based analysis of proteomic data to detect proteins with significant abundance changes
Source: Cell Discov. 2019 Aug 13;5:40. doi: 10.1038/s41421-019-0107-9 (PMC6796874; doi:10.1038/s41421-019-0107-9)
Supplement: Supplementary file 1 — Supplementary information, notes and Figures. [file 41421_2019_107_MOESM1_ESM.pdf]

Supplementary information

MAP: model-based analysis of proteomic data to identify proteins with significant abundance changes

Supplementary Notes

Supplementary note 1: A comparison of different strategies of protein intensity normalization

Supplementary note 2: Discussion of using the Z-statistic defined in MAP to describe protein expression change

Supplementary note 3: Discussion of merging the parallel technical replicates generated in each MS run

Supplementary note 4: Comparing the performance of MAP and other existing tools in differential protein expression analysis based on benchmark differentially expressed proteins

Supplementary note 5: Permutation-based analysis for false discovery rate (FDR) estimation

Supplementary note 6: The extended MAP (eMAP) model for simultaneously comparing multiple proteomic profiles

Supplementary Figures

Supplementary Figure S1-6

Supplementary Tables

Supplementary Table S1-2

Supplementary note 1: A comparison of different strategies of protein intensity normalization

To start a unbiased comparison of two proteomic profiles, the protein intensities in two profiles should first be normalized to make them comparable. In MAP, we proposed a new strategy of protein intensity normalization based on the “trimmed” total intensity of each profile, which was calculated as the sum of protein intensities over proteins that were not identified as outliers in both profiles. In previous studies, usually the total intensity of all detected proteins was used as a reference for global normalization [1, 2]. However, it has been noted in RNA-seq data analysis that genes highly expressed in only one experimental condition may cause sampling artifact and thus could introduce obvious bias to the following comparisons [3]. For example, hemoglobin genes are dramatically upregulated during mammalian erythropoiesis and may comprise a considerable portion of the total transcriptome and proteome at late stages [4], which should be taken into account in the data analysis. Thus, in MAP we label the proteins with extremely high intensities as outliers and exclude them from calculating normalization factors. To test this new strategy, we compared the ratios of protein intensities normalized by the total intensity and by the “trimmed” total intensity of each profile. Similar to what was observed with RNA-seq data [3], the distribution of the $\log_2$-ratios of protein intensities normalized by the total intensity of each profile obviously deviated from zero, and this bias was largely eliminated by using the “trimmed” total intensity instead (Supplementary Fig. S1b).

Next, we further incorporated the ribosome profiling data of undifferentiated and differentiated mESCs to evaluate the performance of two protein intensity normalization strategies. Inspired by the analysis presented in a previous study [5], we picked out the genes that only showed weak translation changes during mESC differentiation from ribosome profiling data (genes with |log2-ratio|<0.2). For these genes, the distribution of their log2-ratios of protein intensities normalized by trimmed total intensity did not show significant deviation from 0 in all three runs (Supplementary Fig. S3c). However, the distribution of their log2-ratios of protein intensities normalized by total

intensity showed a significant deviation from 0 in all three runs (Supplementary Fig. S3c), which is not consistent with the observation from ribosome profiling data for these genes. Then, we separately applied MAP to the protein intensities normalized by two strategies. Interestingly, the top proteins up-regulated in differentiated mESCs detected by using the protein intensities normalized by trimmed total intensity showed obviously higher consistency scores compared to those detected by total intensity-based normalization (Supplementary Fig. S3d). On the other hand, the top proteins up-regulated in undifferentiated mESCs based on these two normalization strategies did not show clear difference in their consistency scores. These findings indicate that normalization of protein intensities simply based on the total intensity may introduce an apparent bias, which could clearly reduce the accuracy of the detection of differentially expressed proteins.

Supplementary note 2: Discussion of using the Z-statistic defined in MAP to describe protein expression change

In many previous studies, it has been shown that the contribution of technical and systematical errors to the log2-ratio of protein intensities observed for each protein depends on the intensity level of this protein [6, 7]. Thus, the raw protein ratios observed in different mass spectrometry (MS) runs may not be directly comparable, since a considerable difference often can be observed between the intensity levels of each protein detected in different runs (Supplementary Fig. S1c). We compared the log2-ratios of protein intensities between the proteomic profiles of undifferentiated and differentiated mESCs generated in different MS runs, and found they showed a low correlation with each other (Supplementary Fig. S2d). On the other hand, the contribution of technical and systematical errors to the Z-statistic defined in MAP can be assumed to follow standard normal distribution. Thus, it should be more comparable across different runs than the raw log-ratios of protein intensities. To support this hypothesis, we also calculated the correlation between the Z-statistics of each protein across different runs, and found they have a much better

correlation (Supplementary Fig. S2e). Thus, the new Z-statistic could be better used to represent protein intensity changes.

Inspired by this finding, we defined the average Z-statistic for each protein to represent its overall intensity change across all comparisons, which is similar to the Z-score defined in Stouffer's Z test [8]. Here we use Lef1 as an example, which has been validated to be differentially expressed at protein level during mESC differentiation by western blot [1]. It can be seen that although Lef1 was only detected to have significant expression change in one MS run (*P*-value=0.005 without correcting for multiple testing), its average Z-statistic over all three runs is still quite significant (*P*-value=0.003 after correcting for multiple testing), as it showed a consistent expression change in all three runs (Fig. S4a). We then tried using the average Z-statistics of proteins to identify proteins differentially expressed between undifferentiated and differentiated mESCs, and found the top differentially expressed proteins identified by this method could also achieve a much improved consistency score compared to those selected simply by the best P-value of three runs (Fig. S4b).

Supplementary note 3: Discussion of merging the parallel technical replicates generated in each MS run

In this study, proteomic profiling of undifferentiated and differentiated mESCs was performed using conventional 4-channel iTRAQ technique. In each MS run, four proteomic profiles were generated, including two parallel technical replicates from iTRAQ channel 114 and 115 for undifferentiated mESCs as well as two parallel technical replicates for differentiated mESCs from channel 116 and 117. Before carrying out the analysis shown in main text, the iTRAQ intensities from channel 114 and 115 were added to generate the proteomic profile of undifferentiated mESCs, and those from channel 116 and 117 were also combined as the proteomic profile of differentiated mESCs. Then, the two combined proteomic profiles of undifferentiated and differentiated mESCs were used as input of MAP model to detect differentially expressed proteins

between them. Meanwhile, we additionally generated two combined proteomic profiles, by averaging the iTRAQ intensities from channel 114 and 116 as well as those from channel 115 and 117, respectively, as two technical replicates used for model fitting using the method proposed in *Zhang et al*.

To illustrate the necessity of this procedure, we repeated the differential protein expression analysis between undifferentiated and differentiated mESCs using the original four proteomic profiles directly obtained from channel 114-117 in each of three MS runs. In this analysis, the proteomic profile of undifferentiated mESCs from channel 114 was compared with the profile of differentiated mESCs from channel 116 using MAP model, and the proteomic profile from channel 115 was compared with the one from channel 117, respectively. Then, we systematically analyzed the protein expression changes detected from these comparisons. Interestingly, the correlation of the Z-statistics derived from different runs in this analysis (Pearson Correlation Coefficients shown in the white boxes of Supplementary Fig. S2f), was found to be clearly lower than that obtained from the combined proteomic profiles (Supplementary Fig. S2e). This finding indicates that combining the iTRAQ intensities from parallel technical replicates generated in the same MS run could suppress the impact of instrumental errors, and thus help to improve the reproducibility of downstream analysis.

Supplementary note 4: Comparing the performance of MAP and other existing tools in differential protein expression analysis based on benchmark differentially expressed proteins

Here, we additionally tried defining benchmark differentially expressed proteins (DEPs) between undifferentiated and differentiated mESCs and using them to evaluate the performance of different methods in differential protein expression analysis. To be fair to all three methods being compared, i.e. MAP, the method proposed in Zhang et al. and MaxQuant, we defined the benchmark up/down-regulated proteins during mESC differentiation as those that were identified

as significantly up/down-regulated proteins (using P-value<0.1 as cutoff) by all three methods in at least two of the three runs, respectively. In this way, we got 206 up-regulated and 110 down-regulated proteins as benchmark DEPs. Then, we checked whether these benchmark DEPs could be efficiently recalled from the comparison of proteomic profiles generated in each single run using the three methods. It can be seen that in all three runs, MAP recalled more benchmark DEPs than the other two methods (Supplementary Fig. S4a-b), indicating it has a better sensitivity in detection of truly differentially expressed proteins.

To fully assess the sensitivity and specificity of three methods, we further examined whether the significance level of each protein's intensity change derived by them from the proteomic data generated in each single run can be used to accurately distinguish the benchmark DEPs from other proteins. It should be note that, the benchmark DEPs were defined in a stringent way. Thus, it's not surprising to find that all three methods achieved a high accuracy in separating benchmark DEPs from the others, with an area under the receiver operating characteristic (ROC) curve (AUC) above 0.9 in all three runs (Fig. S4d). However, we still observed that MAP achieved a higher AUC score than the other two methods in all three runs (Fig. S4d). To test the significance of this difference, we performed 10,000 times of random sampling of non-DEPs and repeated the above analysis. At each time, the benchmark DEPs were matched with 2000 randomly selected other proteins detected in each run, and the AUC score was then recalculated for each of three methods. Again, MAP was found to have a better AUC score compared to the other two methods in classifying the benchmark DEPs against each of the 10,000 sets of randomly selected other proteins (Supplementary Fig. S4c). Taken together, these findings strongly support a favorable performance of our MAP model in differential analysis of iTRAQ proteomic data.

Supplementary note 5: Permutation-based analysis for false discovery rate (FDR) estimation

Here we first describe how we estimated FDR for the second-best *P*-value of each protein, e.g. P_i^{2nd} for protein i, obtained from the comparisons of proteomic data of undifferentiated and differentiated mESCs generated in all three runs, which represents the type-I error rate of the differentially expressed proteins defined by using P_i^{2nd} as cutoff. In previous studies, several permutation-based methods have been proposed for FDR estimation in differential analysis of gene expression microarray data [9, 10]. The key strategy of these methods is that, when estimating FDR for the differentially expressed genes defined by a specific significance cutoff, it's better to include only the non-differentially expressed genes in building the null distribution of test statistic using random permutations. To provide a fast estimation of FDRs for thousands of top-ranked second-best *P*-values, we chose to use a fixed set of non-differentially expressed proteins (non-DEPs) using a specific cutoff, which were defined as proteins with the second-best *P*-values above 0.01, and then performed 10,000 times of random permutations. At each time of random permutation, the Z-statistics of these non-DEPs detected in each run were randomly permuted, and then the second-best *P*-value of each non-DEP was re-calculated. Finally, the estimated FDR for P_i^{2nd} was calculated as

$$FDR\left(P_i^{2nd}\right) = \frac{1}{\pi_0} \times \frac{The\ median\ number\ of\ proteins\ with\ P^{2nd}\ in\ random\ permutations \leq P_i^{2nd}}{Number\ of\ proteins\ with\ P^{2nd}\ observed\ in\ real\ data \leq P_i^{2nd}}$$

, where

$$\pi_0 = \frac{Number\ of\ nonDEPs\ used\ in\ random\ permutations}{Number\ of\ proteins\ detected\ in\ at\ least\ 2\ runs}$$

. To test the effectiveness of this approach, we borrowed the second-best *P*-values $\tilde{P}^{2nd}$ derived from the comparisons of technical replicates generated in three runs using MAP to estimate an FDR for each P_i^{2nd}, which was calculated as

$$FDR^{\#}(P_i^{2nd}) = \frac{Number\ of\ proteins\ with\ \tilde{P}^{2nd} \leq P_i^{2nd}}{Number\ of\ proteins\ with\ P^{2nd} \leq P_i^{2nd}}$$

, and used it as a reference to assess the FDR estimated using the permutation-based approach. We first compared the FDRs estimated from technical replicates with those estimated by random permutations without excluding DEPs, and observed a clear overestimation of FDRs by the permutation-based approach (Supplementary Fig. S5a). Then, by excluding DEPs from random permutations, we found the overestimation of FDRs was greatly reduced, indicating a favorable performance by this approach (Fig. S4c). As the result, it can be obviously seen that the top-ranked DEPs defined based on the second-best P-values have a low FDR (Supplementary Fig. S5b).

Next, we describe how the FDR for the average Z-statistic of each protein, e.g. Z_i for protein i, was estimated, which represents the type-I error rate of differentially expressed proteins defined by using the absolute value of Z_i, i.e. $|\mathrm{Z}_i|$, as cutoff. Similarly, to have a fast estimation of FDRs, in the random permutation analysis we only used a fixed set of non-DEPs, which were defined as proteins with adjusted *P*-values derived from the average Z-statistics above 0.01 here. At each of 10,000 times of random permutations, the Z-statistics of these non-DEPs detected in each run were randomly permuted, and then the average Z-statistic of each non-DEP was re-calculated. Finally, the estimated FDR for Z_i was calculated as

$$FDR(\mathrm{Z}_i) = \frac{1}{\pi_0} \times \frac{The\ median\ number\ of\ proteins\ with\ |\mathrm{Z}|\ in\ random\ permutations \geq |\mathrm{Z}_i|}{Number\ of\ proteins\ with\ |\mathrm{Z}|\ observed\ in\ real\ data \geq |\mathrm{Z}_i|}$$

, where

$$\pi_0 = \frac{Number\ of\ nonDEPs\ used\ in\ random\ permutations}{Number\ of\ all\ detected\ proteins}$$

. Again, to test the effectiveness of this approach, we borrowed the average Z-statistics $\tilde{\mathrm{Z}}$ derived from the comparisons of technical replicates to estimate an FDR for each Z_i, which was calculated as

$$FDR^{\#}(\mathrm{Z_i}) = \frac{Number\ of\ proteins\ with\ average\ Z - statistic\ |\tilde{\mathrm{Z}}| \geq |\mathrm{Z}_i|}{Number\ of\ proteins\ with\ average\ Z - statistic\ |Z| \geq |\mathrm{Z}_i|}$$

. In this way, it can be found that the top-ranked DEPs selected based on average Z-statistics was also associated with a low FDR (Supplementary Fig. S5c), and the FDRs estimated by random permutations generally were supported by those estimated from technical replicates (Supplementary Fig. S5d). Additionally, it is worth to mention that the FDR estimated for the top DEPs ranked by second-best P-values agreed well with that estimated for the same number of top-ranked DEPs selected by average Z-statistics (Supplementary Fig. S5b-c), although they were derived from independent permutation analysis and were calculated using different formula. Since the vast majority of top-ranked DEPs selected by the second-best P-values were also identified as top DEPs based on average Z-statistics (for example, 393 proteins were found to have second-best P-values<0.01, and 380 of them were also found to have adjusted P-values calculated from average Z-statistics lower than 0.01; see Supplementary Table S1 for details), we think this observation is quite reasonable. It suggests that controlling the FDR level, if it's properly estimated, could be a practical and robust way to determine the cutoff of statistical significance in differential expression analysis, as indicated by quite a number of previous studies [11].

Supplementary note 6: The extended MAP (eMAP) model for simultaneously comparing multiple proteomic profiles

It's often needed to perform differential protein expression analysis across multiple cellular contexts. As a natural extension of our original MAP model for pairwise comparisons, we additionally developed a new statistical framework, termed extended MAP (eMAP) model, for simultaneously comparing multiple proteomic profiles generated in the same MS run and directly detecting proteins/peptides with significant intensity changes across these profiles.

Similar to traditional Analysis of Variance (ANOVA), in eMAP we assume that, for every detected protein i, it has equal abundance/expression level among the samples being compared, which is used as the null hypothesis. Thus, the log2-transformed intensities of this protein in the N input proteomic profiles can be considered as drawn from the same normal distribution

$N(\mu_i, \sigma_i^2)$. Here μ_i indicates the expected intensity level of this protein and σ_i^2 is used to represent the impact of technical and systematic errors (or noise), and is empirically modeled as a function of its intensity level, which is the global variance function that needs to be inferred from input data.

To directly build global variance function from the N proteomic profiles being compared, we adopt a similar step-by-step regression procedure as that used in our original MAP model. Here we use S_i^j to denote the log2-transformed intensity of protein i in the j-th input profile, $\bar{S_\iota} = \sum_{j=1}^{N} S_i^j / N$ denotes its mean log2-intensity over the N input profiles, and

$$V_i = \frac{\sum_{j=1}^{N} (S_i^j - \bar{S_\iota})^2}{N-1}$$

denotes the observed sample variance of its log2-intensities. Based on Cochran's theorem [12], we have

$$V_i * \frac{N-1}{\sigma_i^2} = \frac{\sum_{j=1}^{N} (S_i^j - \bar{S_\iota})^2}{\sigma_i^2} \sim \chi_{N-1}^2$$

, where χ_{N-1}^2 is the Chi-square distribution with *N-1* degrees of freedom. In eMAP, σ_i^2 is modeled as a function of $\bar{S_\iota}$ in the form $\sigma^2 = \Psi(\boldsymbol{\theta}, \bar{S}) = \exp(\theta_1 + \theta_2 \cdot \bar{S}) + \theta_3$, i.e. the so-called global variance function, and the values of parameters $\boldsymbol{\theta}$ would be directly inferred from the proteomic profiles being compared. Different from that suggested by *Zhang et al* [7] for pairwise comparison of proteomic profiles, in eMAP we do not always assume $\theta_3 = 0$, as we found that sometimes the observed variance of log2-protein intensities across multiple proteomic profiles may not asymptotically drop to 0 for proteins with high intensity levels. Then, by using the sample variance V_i of each protein's log2-intensities and Chi-square distribution χ_{N-1}^2 to replace the log2-ratio of protein intensities and standard normal distribution $N(0,1)$ used in the local linear regression analysis of our original MAP model, respectively, we finally have the entire statistical framework of eMAP model to infer the values of parameters $\boldsymbol{\theta}$ of global variance function $\sigma^2 = \Psi(\boldsymbol{\theta}, \bar{S})$. Technically, eMAP first plots the sample variance of each protein's log2-intensities against its

mean log2-intensity, which we called VA plot, and this plot is then scanned by a sliding window of size N proteins moving from left to right ($N = 400$ and step size=100 by default). In this way, the whole VA plot is covered by a series of windows, which are used to first derive the local approximations of global variance function. Again, eMAP introduces an assumption that proteins falling in each window have similar intensity levels, and thus the impact of technical and systematic errors to their log2 intensities can be approximately quantified by the same variance parameter σ^2. To estimate the σ^2 for each window, the observed sample variance of all the proteins in this window are ordered from smallest to largest, and then plotted against the corresponding theoretical quantiles $\widehat{q_i}$ of Chi-square distribution χ^2_{N-1} divided by $N-1$. Here, the plotting position $\hat{p}_i$ associated with the i-th sample variance is calculated based on the same formula as that used in MAP. Next, an ordinary least-square linear regression is applied to the smallest W ($W = 30\%$ by default) of the ordered sample variances in each window against the corresponding theoretical quantiles of Chi-square distribution to derive a linear model

$$\mathrm{V} = \sigma^2 * \frac{\hat{q}}{N-1}$$

, and the slope is used to as an estimation of the variance parameter σ^2 for this window. When the sliding window finishes scanning the VA plot, non-linear least-square regression is applied to fit a exponential function between the variance σ^2 estimated for each window and the average of the mean log2-intensities over proteins in this window that were used for linear regression as

$$\sigma^2 = \Psi(\boldsymbol{\theta}, \bar{S}) = \exp(\theta_1 + \theta_2 \cdot \bar{S}) + \theta_3$$

, which is then used to infer the σ_i^2 for every detected protein by $\sigma_i^2 = \exp(\theta_1 + \theta_2 \cdot \overline{S_i}) + \theta_3$. Finally, a Chi-square statistic Q_i is defined for each protein by using the σ_i^2 inferred for it to rescale the observed sample variance of its log2 intensities as

$$Q_i = V_i * \frac{N-1}{\sigma_i^2} = V_i * \frac{N-1}{\Psi(\boldsymbol{\theta}, \overline{S_i})}$$

, which is expected to follow Chi-square distribution χ^2_{N-1} under the null hypothesis, and a *P*-value is calculated for this protein as

$$P_i = \int_{\mathrm{Q}_i}^{+\infty} \chi^2_{N-1}\, dx = \int_{\mathrm{Q}_i}^{+\infty} \frac{\mathrm{x}^{\frac{N-1}{2}-1}\mathrm{e}^{-\frac{x}{2}}}{2^{\frac{N-1}{2}}\Gamma(\frac{N-1}{2})} dx$$

($\Gamma(\cdot)$ denotes the gamma function) to describe the statistical significance of the observed sample variance of its log2 intensities across the proteomic profiles being compared.

Furthermore, usually proteomic profiling experiments are repeatedly performed for multiple times as biological replicates. Again, users can use eMAP to perform a two-step differential analysis on their own proteomic data with replicates generated in different MS runs. At the first step, they can use eMAP to separately compare the proteomic profiles generated in each run. Then, the outputs of these separate comparisons should be combined to finally determine the differentially expressed proteins. We defined the average Chi-square statistic of protein i across k separate comparisons as

$$\bar{Q_\iota} = \sum_{t=1}^{k} Q_i^t / k$$

. Under the null hypothesis, $\bar{Q}$ is expected to follow the Gamma distribution

$$\bar{Q} \sim \Gamma\left(\alpha = \frac{k(N-1)}{2}, \beta = k/2\right) = \frac{(\frac{k}{2})^{\frac{k(N-1)}{2}} \mathrm{x}^{\frac{k(N-1)}{2}-1} \mathrm{e}^{-\frac{xk}{2}}}{\Gamma(\frac{k(N-1)}{2})}$$

, where $\Gamma(\alpha, \beta)$ denotes the gamma distribution with shape parameter α and inverse scale parameter β. Next, a *P*-value is calculated for the average Chi-square statistic of protein i as

$$P_i = \int_{\bar{Q_\iota}}^{+\infty} \Gamma\left(\frac{k(N-1)}{2}, \frac{k}{2}\right) dx = \int_{\bar{Q_\iota}}^{+\infty} \frac{\left(\frac{k}{2}\right)^{\frac{k(N-1)}{2}} \mathrm{x}^{\frac{k(N-1)}{2}-1} \mathrm{e}^{-\frac{kx}{2}}}{\Gamma\left(\frac{k(N-1)}{2}\right)} dx$$

, which will be further adjusted using the Benjamini-Hochberg approach for multiple testing. It should be of note that, all the *P*-values define in this section are one-tailed *P*-values, which

represent the likelihood of observing an equal or greater sample variance of log2 protein intensities compared to that expected under the null hypothesis.

To test the validity of eMAP model, we applied it to compared the original four proteomic profiles of undifferentiated and differentiated mESCs generated in each of the three runs. Again, here we use the comparison of four proteomic profiles generated in the first MS run to illustrate the workflow of eMAP. First, we generated a VA plot by plotting the sample variance of each protein's log2-intensities against its mean log2-intensity (Supplementary Fig. S6a), and then used a sliding window moving from left to right to scan the VA plot. Of note, proteins in the window should be considered as a mixture of differentially and non-differentially expressed proteins. To directly infer the impact of technical and systematic errors from the non-differentially expressed proteins in the mixture, the observed sample variances of all proteins in this window were ordered from smallest to highest and then plotted against the corresponding theoretical quantiles of the Chi-square distribution χ_3^2 divided by 3. It can be seen that the ordered sample variances located on the left side, which could be assumed to be mainly associated with non-differentially expressed proteins, exhibited a strong linear relationship with the corresponding theoretical quantiles (right panel of Supplementary Fig. S6a). Then, an ordinary least-square linear regression analysis was applied between the smallest W of the sample variances and the corresponding theoretical quantiles (we chose W=30% here, as it's difficult to expect a very large fraction of the detected proteins are non-differentially expressed between any possible pair of four input proteomic profiles), and a liner model with very high coefficient of determination was derived (right panel of Supplementary Fig. S6a). The slope σ^2 of the fitted linear model can be used to as a local approximation of the global variance function for proteins covered by this window.

As the sliding window moving in a stepwise manner from left to right, the whole VA plot was covered by a series of windows. For each window, the same linear regression analysis was repeatedly applied to estimate the σ^2 as a local approximation of the global variance function.

Remarkably, the coefficient of determination R^2 was found to be higher than 0.95 for all linear regressions, strongly supporting the validity of our approach. Next, the σ^2 inferred for each window was plotted against the mean $\log_2$-protein intensity averaged over proteins falling in this window, and an exponential function was fitted between the variance and the mean with coefficient of determination R^2=0.994 (Supplementary Fig. S6b). This exponential function is right the global variance function we want to infer for the four profiles under comparison. Finally, a *P*-value was calculated for each protein to describe the significance of the variation of its intensities across the four input proteomic profiles (Supplementary Fig. S6c).

Similar to what was done in MAP, an additional analysis was also carried in eMAP to summarize all the local linear regressions. In this analysis, the ordered sample variances in each window were rescaled by the σ^2 inferred for this window. Then, the mean and standard deviation of the rescaled sample variances across all windows were plotted according to their order against the corresponding theoretical quantiles of Chi-square distribution χ_3^2 divided by 3 (Supplementary Fig. S6d). This analysis could be used to inspect whether the parameter W needs to be further adjusted. Here we found that, after rescaling, the smallest 30% of the sample variances were found to match with the line y=x very well. Meanwhile, the ordered sample variances on the right side obviously deviated from line y=x, which suggests a considerable proportion of them are difficult to be explained by the null hypothesis and may actually be associated with proteins differentially expressed between undifferentiated and differentiated mESCs. Thus, they should not be included in inferring the local and global variance function.

After separately applying eMAP to compare the proteomic profiles of undifferentiated and differentiated mESCs generated in each of three runs, we collected the output from all three runs, and identified proteins with significantly elevated variances of protein intensities using *P*-values calculated based on the average Chi-square statistic of each protein over three runs after adjusting for multiple testing, which we called hyper-variable proteins (HVPs). In total, we obtained

383 HVPs with adjusted *P*-values lower than 0.01 (Supplementary Table S2), the vast majority of which were also identified as differentially expressed proteins (DEPs) between undifferentiated and differentiated mESCs by using MAP model (Supplementary Fig. S6e), indicating a high consistency between these two analyses. Furthermore, by incorporating the ribosomal profiling data of undifferentiated and differentiated mESCs, we found that the RNA transcripts from genes associated with the 383 HVPs showed a significantly higher change of ribosome occupancy during the differentiation of mESCs than the other genes detected in ribosomal profiling, indicating these genes tend to be differentially translated (Supplementary Fig. S6f). Taken together, these findings indicate that our eMAP model can be used to statistically model the variations of protein intensities across multiple proteomic profiles and reliably identify proteins with significant expression changes among the profiles being compared.

Reference:

1. Zhou, F., et al., *Genome-scale proteome quantification by DEEP SEQ mass spectrometry.* Nat Commun, 2013. **4**: p. 2171.
2. Kim, M.S., et al., *A draft map of the human proteome.* Nature, 2014. **509**(7502): p. 575-81.
3. Robinson, M.D. and A. Oshlack, *A scaling normalization method for differential expression analysis of RNA-seq data.* Genome Biol, 2010. **11**(3): p. R25.
4. Liu, X., et al., *Regulation of mitochondrial biogenesis in erythropoiesis by mTORC1-mediated protein translation.* Nat Cell Biol, 2017. **19**(6): p. 626-638.
5. Shao, Z., et al., *MAnorm: a robust model for quantitative comparison of ChIP-Seq data sets.* Genome Biol, 2012. **13**(3): p. R16.
6. Breitwieser, F.P., et al., *General statistical modeling of data from protein relative expression isobaric tags.* J Proteome Res, 2011. **10**(6): p. 2758-66.
7. Zhang, Y., et al., *A robust error model for iTRAQ quantification reveals divergent signaling between oncogenic FLT3 mutants in acute myeloid leukemia.* Mol Cell Proteomics, 2010. **9**(5): p. 780-90.
8. Whitlock, M.C., *Combining probability from independent tests: the weighted Z-method is superior to Fisher's approach.* Journal of Evolutionary Biology, 2005. **18**(5): p. 1368-1373.

9. Xie, Y., W. Pan, and A.B. Khodursky, *A note on using permutation-based false discovery rate estimates to compare different analysis methods for microarray data.* Bioinformatics, 2005. **21**(23): p. 4280-8.
10. Jiao, S. and S. Zhang, *On correcting the overestimation of the permutation-based false discovery rate estimator.* Bioinformatics, 2008. **24**(15): p. 1655-61.
11. Storey, J.D. and R. Tibshirani, *Statistical methods for identifying differentially expressed genes in DNA microarrays.* Methods Mol Biol, 2003. **224**: p. 149-57.
12. Cochran, W., *The distribution of quadratic forms in a normal system, with applications to the analysis of covariance.* Mathematical Proceedings of the Cambridge Philosophical Society, 1934. **30**(2): p. 178-191.

Supplementary Figures

Supplementary Figure S1

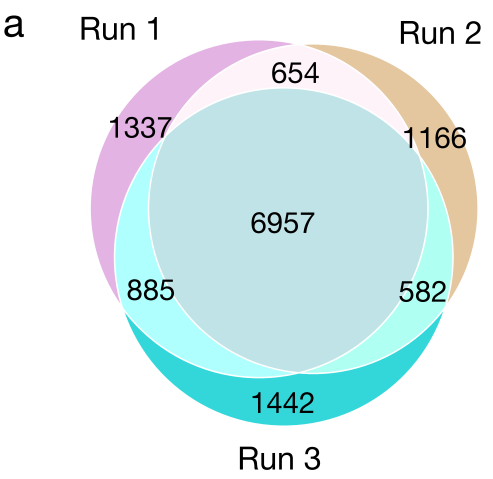

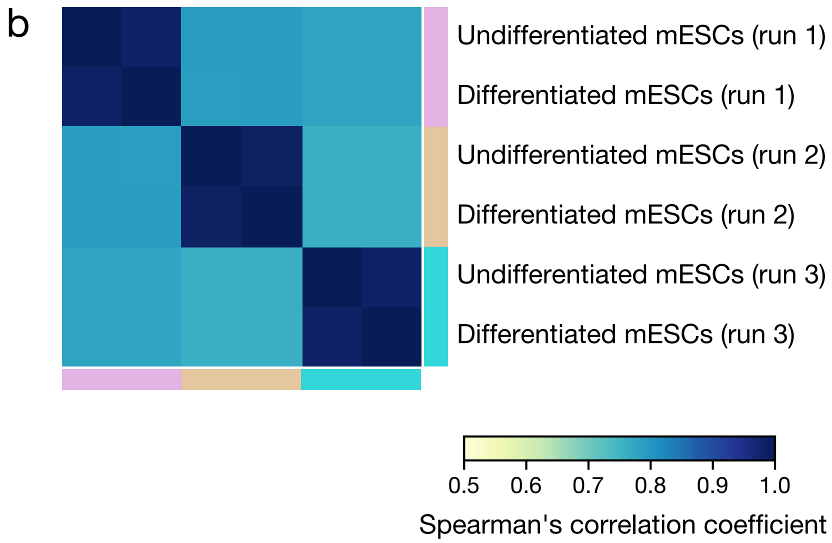

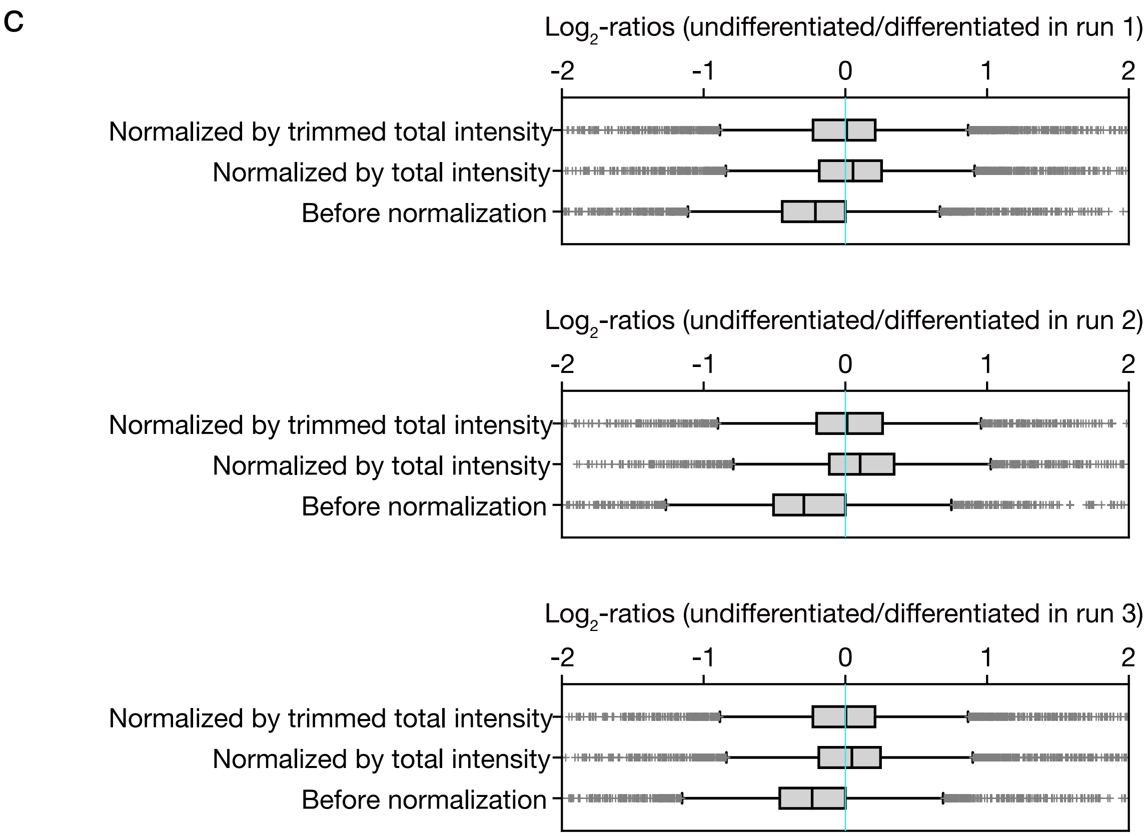

Supplementary Figure S1: Quantitative proteomic profiling of undifferentiated and differentiated mESCs using iTRAQ technique.

(**a**) Venn diagram showing overlap of the total proteins detected in each MS run. (**b**) Boxplot of the log_2-ratios of protein intensities for all the proteins detected in each run. Here two different strategies of iTRAQ intensity normalization were used: normalization based on the total intensity or the trimmed total intensity of each proteomic profile. The log_2-ratios of raw protein intensities without any normalization were also plotted as a reference. (**c**) Correlation analysis of the protein intensities across the proteomic profiles of undifferentiated and differentiated mESCs generated in all three MS runs as biological replicates. Here the correlation was quantified by Spearman's correlation coefficient.

Supplementary Figure S2

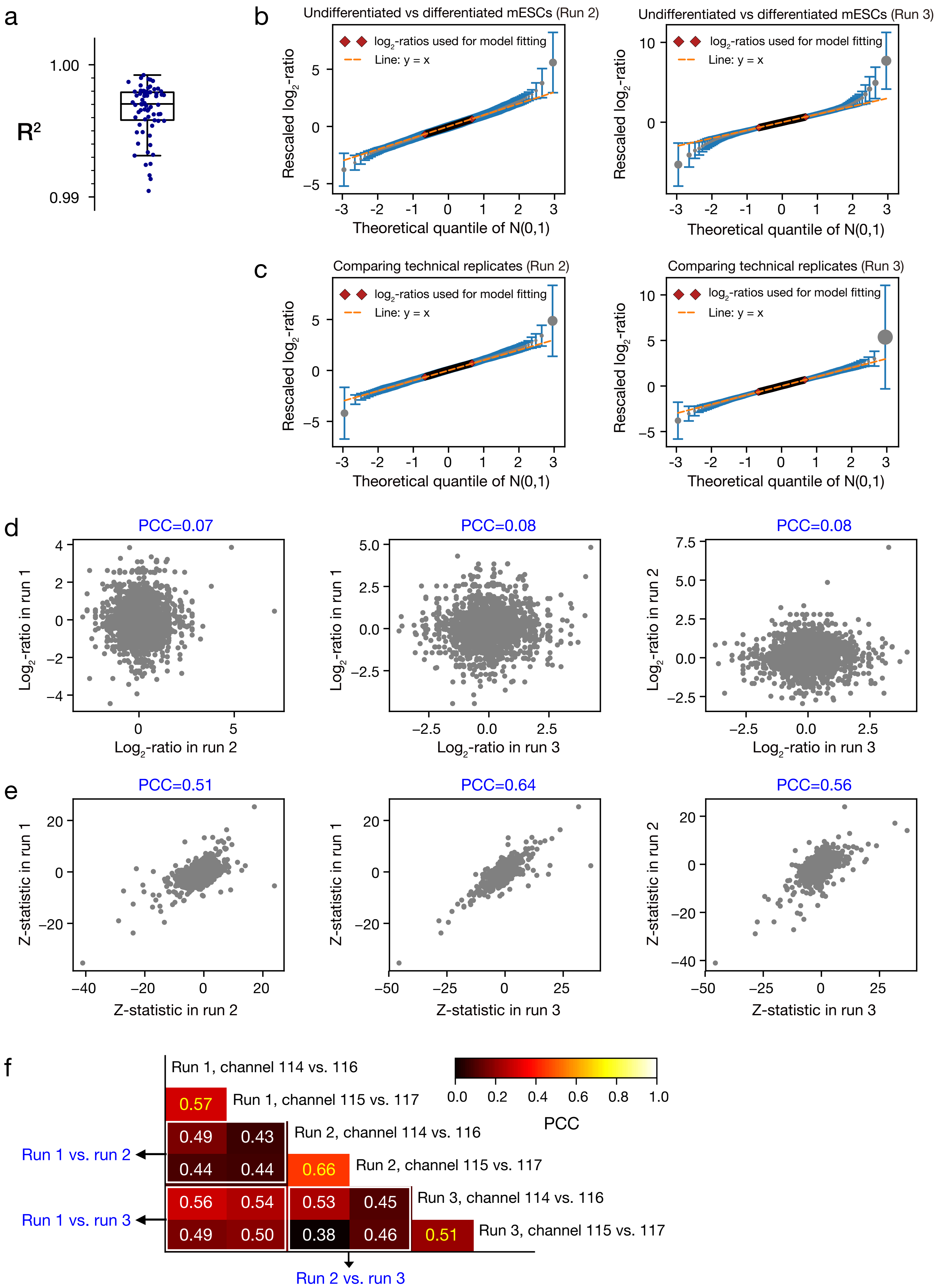

Supplementary Figure S2: Using MAP to compare technical replicates.

(**a**) R^2 values of all the linear regressions when the sliding window shown in Fig. 2a finished scanning the whole plot. (**b**) The same plot as Figure 2D, but here MAP was applied to compare the proteomic profiles of undifferentiated and differentiated mESCs generated in the second and the third run. (**c**) The same plot as Figure 2E, but here MAP was applied to compare the technical replicates generated in the second and the third run. (**d**) Scatter plot of the $\log_2$-ratios of protein intensities between the proteomic profiles of undifferentiated and differentiated mESCs generated in different MS runs. Here Pearson's correlation coefficient (PCC) between the $\log_2$-ratios of each protein across different runs was also calculated. (**e**) Scatter plot of the Z-statistics of protein intensity changes between the proteomic profiles of undifferentiated and differentiated mESCs generated in different MS runs. Here PCC between the Z-statistics of each protein across different runs was also calculated. (**f**) Heatmap to show the PCC of the Z-statistics of protein intensity changes between undifferentiated and differentiated mESCs detected in different comparisons. Here the pairwise PCCs may either be calculated between two separate comparisons using proteomic profiles from the same MS run but different channels, or using those from different runs.

Supplementary Figure S3

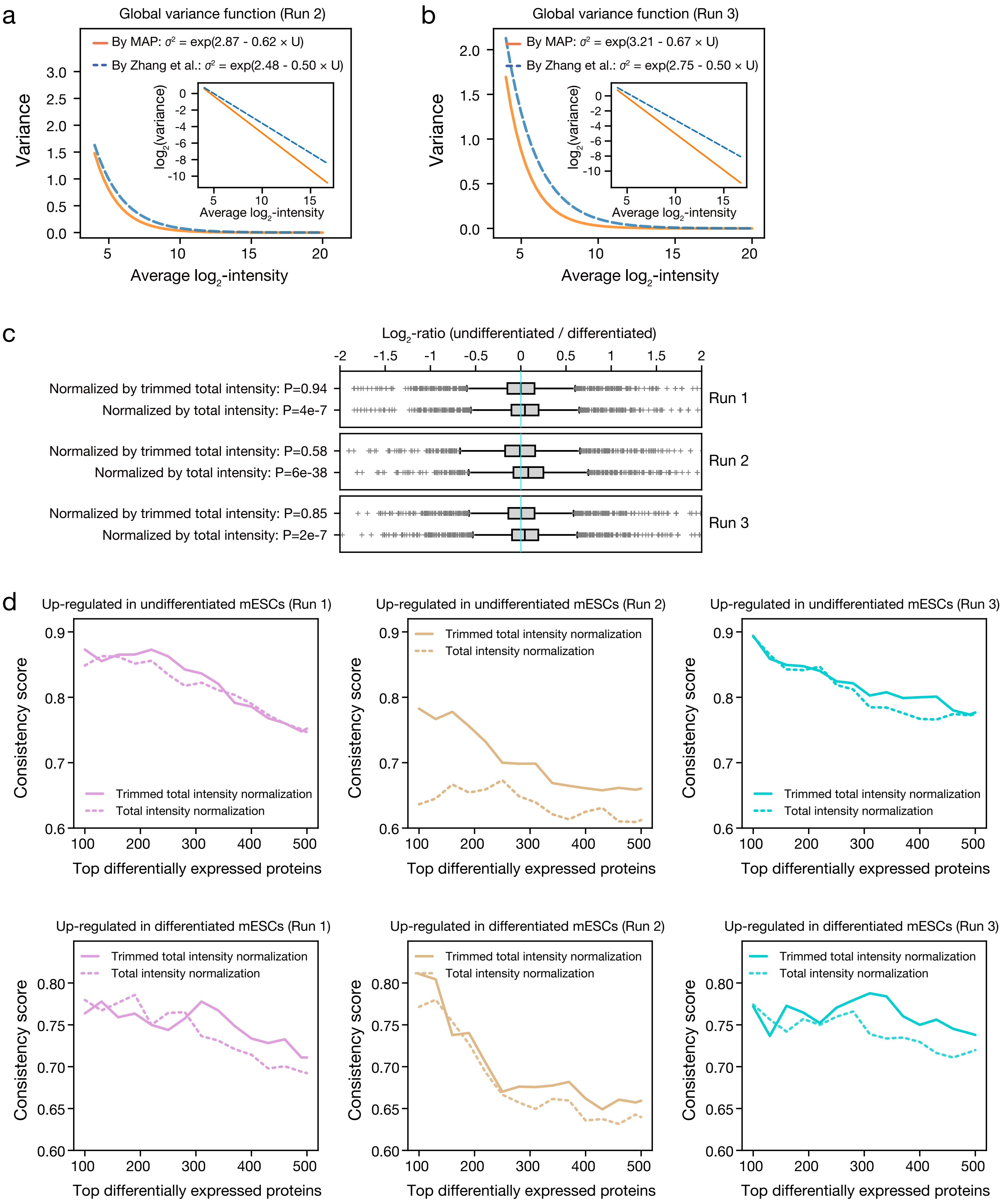

Supplementary Figure S3: Comparison of the global variance functions inferred by MAP and the method proposed in Zhang et al.

(**a**-**b**) The global variance function obtained by using MAP to compare the proteomic profiles of undifferentiated and differentiated mESCs generated in the second (**a**) and the third (**b**) run (solid line), as well as that obtained by applying the method proposed in Zhang *et al* on the technical replicates generated in the same run (dashed line). (**c**) Boxplot of the $\log_2$-ratios of protein intensities either normalized by the total intensity or by the trimmed total intensity of each profile. Here only the proteins show weak translation changes in the ribosome profiling experiment ($|\log_2\text{-ratio}|<0.2$) were used and the *P*-values were calculated by using one-sample Student's t-test to compare each group of $\log_2$-ratios against 0. (**d**) Comparison of the consistency score of the top up-regulated proteins in undifferentiated (upper panels) and differentiated (lower panels) mESCs based on protein intensities normalized by the total intensity (solid line) as well as by the trimmed total intensity (dashed line) of each profile. Here proteins were ranked by their P-values of intensity changes calculated by MAP.

Supplementary Figure S4

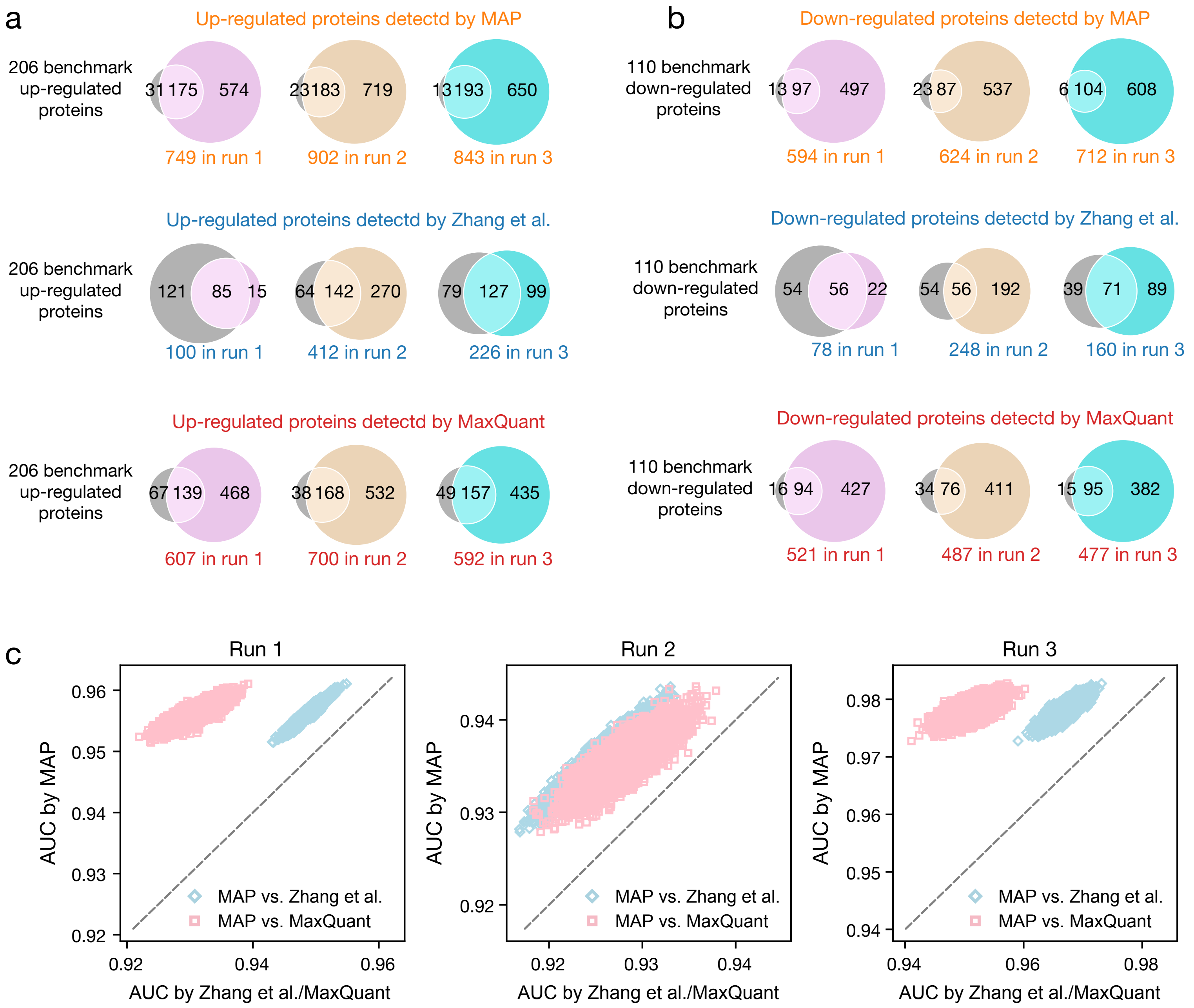

Supplementary Figure S4: MAP can accurately recall the benchmark differentially expressed proteins between undifferentiated and differentiated mESCs using proteomic data generated in each single run

(**a**) Venn diagrams to show the overlap between the benchmark up-regulated proteins during mESC differentiation and the significantly up-regulated proteins identified by MAP, the method proposed by Zhang et al. and MaxQuant from comparing the proteomic profiles generated in each single run (using P-value<0.05 as cutoff). Here the benchmark up-regulated proteins were defined as those that were identified as significantly up-regulated proteins by all three methods in at least two of three runs. (**b**) Venn diagrams to show the overlap between the benchmark down-regulated proteins during mESC differentiation and the significantly down-regulated proteins identified by MAP, the method proposed by Zhang et al. and MaxQuant from comparing the proteomic profiles generated in each single run (using P-value<0.05 as cutoff). Here the benchmark down-regulated proteins were defined as those that were identified as significantly down-regulated proteins by all three methods in at least two of three runs. (**c**) Scatter plot of the AUC (area under the receiver operating characteristic curve) value achieved by using the P-values derived by MAP in each single run to distinguish the benchmark DEPs versus 2000 randomly selected other proteins against that achieved by the method proposed by Zhang et al./MaxQuant. Here the random sampling of other proteins was repeated for 10000 times. The dashed lines indicate the line y=x.

Supplementary Figure S5

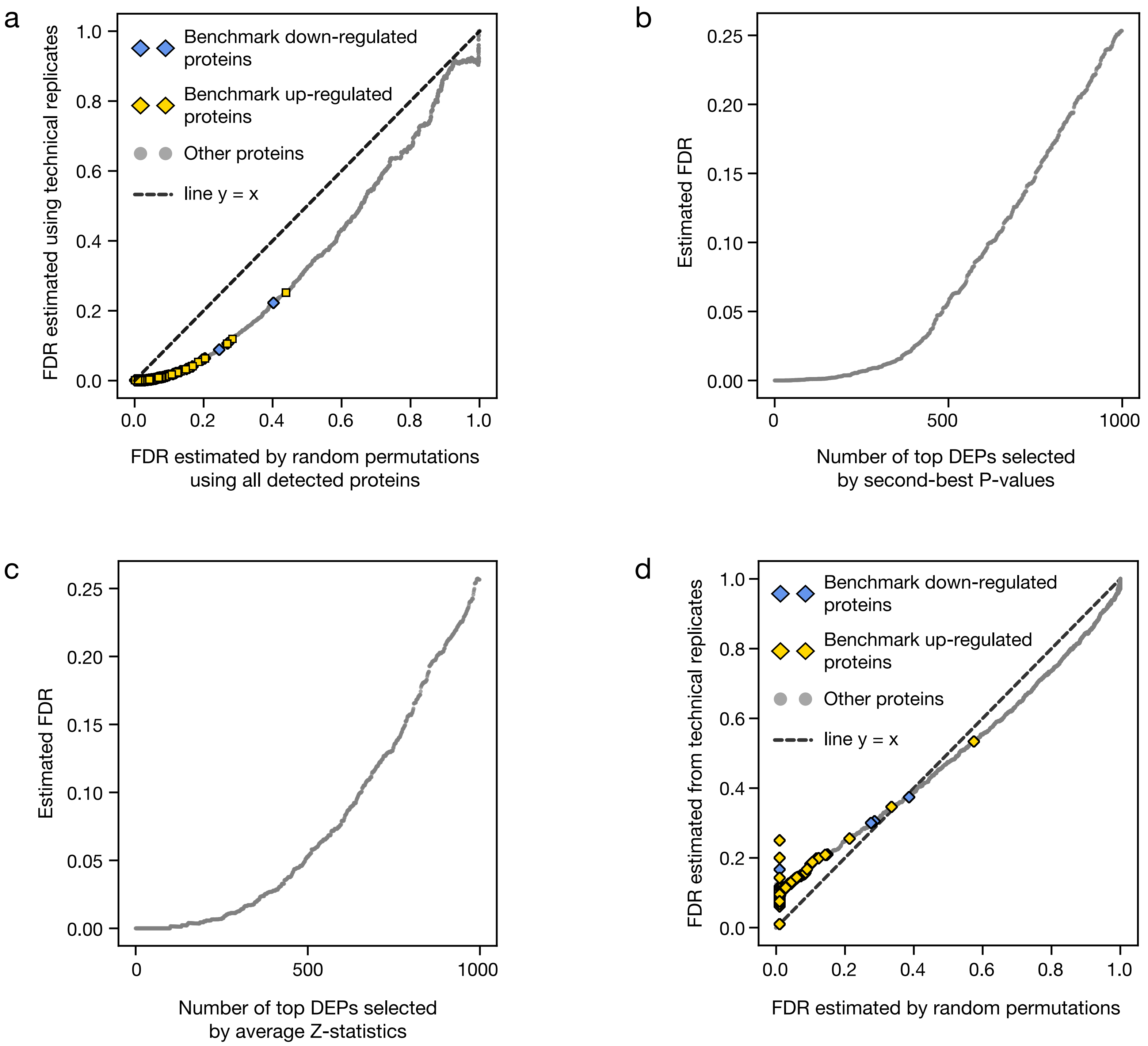

Supplementary Figure S5: Comparing the false discovery rates (FDRs) estimated using random permutations and those estimated from the technical replicates.

(**a**) False discovery rate (FDR) estimated for the second best P-value of each protein using the permutation-based approach without excluding the differentially expressed proteins were plotted against that estimated from the comparisons of technical replicates for this second best P-value. Here FDRs associated with the benchmark DEPs defined previously were explicitly indicated. (**b**-**c**) False discovery rate (FDR) estimated for the top differentially expressed proteins (DEPs) using the permutation-based approach. Here the top DEPs were selected based on the second-best P-value (**b**) or the average Z-statistic (**c**) achieved by each protein, respectively. (**d**) False discovery rate (FDR) estimated for the average Z-statistic of each protein using the permutation-based approach were plotted against that estimated from the comparisons of technical replicates for this average Z-statistic. Here FDRs associated with the benchmark DEPs defined previously were explicitly indicated

Supplementary Figure S6

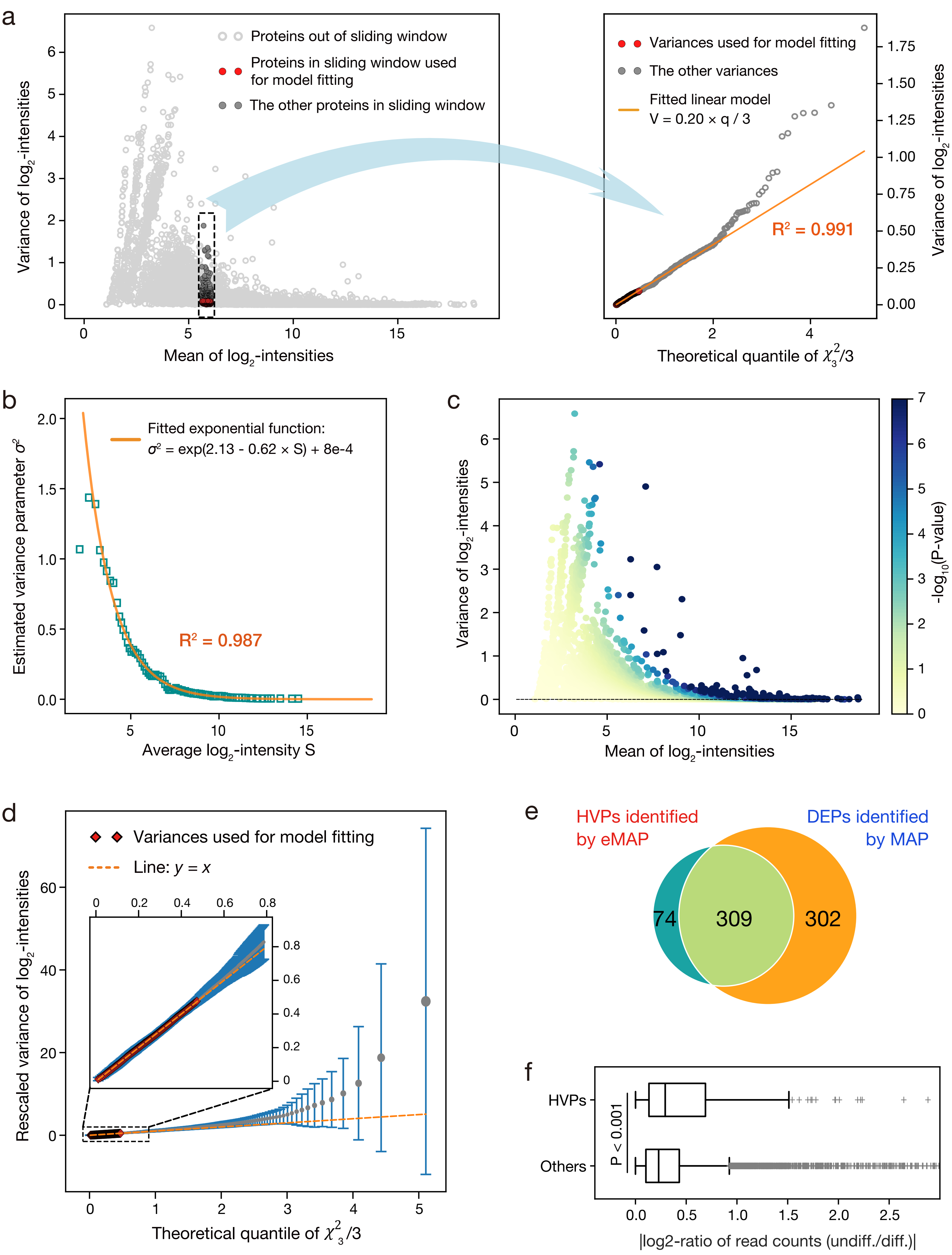

Supplementary Figure S6: Using eMAP to compare the original four proteomic profiles of undifferentiated and differentiated mESCs generated in each of three runs.

(**a**) VA plot to show the comparison of the four original proteomic profiles of undifferentiated (channel 114 and 115 as technical replicates) and differentiated (channel 116 and 117 as technical replicates) mESCs generated in the first run (left panel). Here x axis is the mean $\log_2$-intensity of each protein among four profiles and y axis is the sample variance of its $\log_2$-intensities. A sliding window of size 400 was used to scan the plot with a step size of 100. At each step, all the variances of proteins covered by the window were ordered from smallest to highest and plotted against the corresponding theoretical quantiles of Chi-square distribution χ_3^2 divided by 3 (right panel). Next, standard least square linear regression was applied to the smallest 30% of the ordered sample variances, which were assumed to be predominantly associated with non-differentially expressed proteins, against the corresponding theoretical quantiles to derive a liner model, and the slope σ^2 of this linear model was taken as an local approximation of the global variance function parameter (right panel). Here R^2 is the coefficient of determination of liner regression. (**b**) The exponential function fitted between the variance parameter σ^2 estimated for each window and the average $\log_2$-intensities of proteins falling in this window. (**c**) The VA plot shown in (**a**) with color coding to indicate the *P*-value of each protein's intensity change across the four proteomic profiles being compared. (**d**) Plot of the ordered variances of log2-protein intensities, which were rescaled by the σ^2 estimated for each window and then averaged across all windows, against the corresponding theoretical quantiles of Chi-square distribution χ_3^2 divided by 3. Error bars represent the standard deviation from the mean. (**e**) Venn diagram illustrating the overlap between the hyper-variable proteins (HVPs) detected by eMAP model (with adjusted *P*-values calculated based on the average Chi-square statistic of three runs lower than 0.01) between undifferentiated and differentiated mESCs and the differentially expressed proteins (DEPs) detected by MAP model (with adjusted *P*-values calculated based on the average Z-

statistic of three runs lower than 0.01). (**f**) Boxplot to show the distribution of the ribosome occupancy changes of genes associated with the 383 HVPs and the other genes during mESC differentiation. Here the ribosome occupancy change of each genes is quantified by the absolute value of the log2-ratio of its normalized ribosome profiling read counts between undifferentiated and differentiated mESCs .

Supplementary Table S1: Tables to show the iTRAQ intensities in undifferentiated and differentiated mESCs for all the proteins detected in each MS run, the log-ratios of their normalized protein intensities between differentiated and undifferentiated mESCs, the corresponding Z-statistics and P-values calculated by MAP, and also the log-ratios of their protein intensities between the technical replicates generated in each MS run together with the corresponding P-values. In the last table, the best P-value, the second best P-value, the average Z-statistic and the corresponding P-value of each protein across all three runs are also included.

Supplementary Table S2: Tables to show the statistics derived by using eMAP to compare the original 4 proteomic profiles of undifferentiated and differentiated mouse mESCs generated in each of three runs. They include the sample variance of each protein's log2-intensities across the 4 proteomic profiles, the corresponding Chi-square (χ^2) statistic and *P*-value of this protein calculated by eMAP in each run, as well as the average Chi-square (χ^2) statistic and corresponding P-value of each protein derived by combining the output of eMAP from all three runs.
